# Hidden dynamics of soccer leagues: The predictive ‘power’ of partial standings

**DOI:** 10.1371/journal.pone.0225696

**Published:** 2019-12-18

**Authors:** Clive B. Beggs, Alexander J. Bond, Stacey Emmonds, Ben Jones

**Affiliations:** 1 Institute for Sport, Physical Activity and Leisure, School of Sport, Leeds Beckett University, Leeds, West Yorkshire, England, United Kingdom; 2 Yorkshire Carnegie Rugby Union club, Leeds, England, United Kingdom; 3 England Performance Unit, The Rugby Football League, Leeds, England, United Kingdom; 4 Leeds Rhinos Rugby League club, Leeds, England, United Kingdom; 5 School of Science and Technology, University of New England, Armidale, New South Wales, Australia; 6 Division of Exercise Science and Sports Medicine, Department of Human Biology, Faculty of Health Sciences, the University of Cape Town and the Sports Science Institute of South Africa, Cape Town, South Africa; Queen Mary University of London, UNITED KINGDOM

## Abstract

**Objectives:**

Soccer leagues reflect the partial standings of the teams involved after each round of competition. However, the ability of partial league standings to predict end-of-season position has largely been ignored. Here we analyze historical partial standings from English soccer to understand the mathematics underpinning league performance and evaluate the predictive ‘power’ of partial standings.

**Methods:**

Match data (1995–2017) from the four senior English leagues was analyzed, together with random match scores generated for hypothetical leagues of equivalent size. For each season the partial standings were computed and Kendall’s normalized tau-distance and Spearman r-values determined. Best-fit power-law and logarithmic functions were applied to the respective tau-distance and Spearman curves, with the ‘goodness-of-fit’ assessed using the R^2^ value. The predictive ability of the partial standings was evaluated by computing the transition probabilities between the standings at rounds 10, 20 and 30 and the final end-of-season standings for the 22 seasons. The impact of reordering match fixtures was also evaluated.

**Results:**

All four English leagues behaved similarly, irrespective of the teams involved, with the tau-distance conforming closely to a power law (R^2^>0.80) and the Spearman r-value obeying a logarithmic function (R^2^>0.87). The randomized leagues also conformed to a power-law, but had a different shape. In the English leagues, team position relative to end-of-season standing became ‘fixed’ much earlier in the season than was the case with the randomized leagues. In the Premier League, 76.9% of the variance in the final standings was explained by round-10, 87.0% by round-20, and 93.9% by round-30. Reordering of match fixtures appeared to alter the shape of the tau-distance curves.

**Conclusions:**

All soccer leagues appear to conform to mathematical laws, which constrain the league standings as the season progresses. This means that partial standings can be used to predict end-of-season league position with reasonable accuracy.

## Introduction

For all the complexities of the game itself, how the outcome of soccer matches are recorded and evaluated is a relatively simple process, with most teams belonging to a league that reflects the standings of the respective clubs after each round of competition. In a typical soccer league teams are awarded three points for a win, one point for a draw and no points for loosing, with the respective partial standings (i.e. the standings part way through the season) at any point in time based on the number of points accumulated. The only variation to this simple rule comes when teams tie on the number of points accumulated. In Bundesliga (Germany), Ligue 1 (France) and the English leagues when ties occur, the difference between the total number of goals scored and conceded is used to ‘fine tune’ the ranking order of the teams, whereas in other leagues, such as La Liga (Spain), ties may be settled using the head-to-head goal difference or points depending on the number of teams involved in the tie. Although much money, time and effort is expended by club owners, coaches, players, pundits and fans considering strategy and tactics in an attempt to predict and influence the outcome of matches, it is often forgotten that in a league the only outcome that ultimately matters is the final position at the end of the season. Although only one team can win the league, the top two or three teams (it varies from league to league) may be promoted to a higher division, or selected to compete in an international competition, such as the *Union of European Football Associations* (UEFA) Champions League, while those in the bottom three or four places are generally relegated to a lower division. Other variations include the introduction of ‘play-off’ positions for the tranche of teams just outside the automatic promotion positions, such as used in the English Championship and English leagues one and two. As such, it is the final league standing, rather than the points accumulated *per se*, that is the measure by which teams and their managers are judged. Indeed, given the financial implications, it is no surprise that the final standings matter. In the English Premier League, for example, each additional placing in the final standings is worth £1.9 million (2017 data) [1], no matter the magnitude of the points differences between adjacent teams. Furthermore, when it comes to issues of promotion and relegation the financial implications may be immense, with, in some cases, relegated clubs forced into liquidation [2].

Given the huge implications (both financial and reputational) of final league standing, it perhaps comes as no surprise that many stakeholders (e.g. clubs, coaches, fans, bookmakers, gamblers, amongst others) have a keen interest in predicting final league position. After all, if club owners and executive decision-makers can predict with some confidence, early in a season, the likely final position of their team, then they may be able to make adjustments to accommodate or avert a negative outcome, as well as setting realistic targets and performance indicators. Indeed many people, particularly those associated with the gambling industry, have developed methodologies for ranking sporting teams and predicting team performance. While many of these methodologies remain unpublished because of confidentiality issues, others are in the public domain. For example, well established mathematical techniques such as the Colley [3–5] and Keener [4–6] algorithms aim to rank teams based on past performance in fractured competition, while techniques such as the Pythagorean expectation system [7, 8] attempt to predict future league performance based on past performance. Still other techniques, such as the Bradley-Terry [9, 10] and Elo [4, 11] methods, seek to predict the outcome of single matches using a probabilistic methodology. The Bradley-Terry and Elo methods have also been adapted for use as ranking systems [11, 12]. In this capacity, the Bradley-Terry and Elo systems consider only the outcome of the matches played when rating teams, whereas the Keener and Colley methods utilize respectively, the total number of scores for and against, and the number of wins and losses achieved, when determining rankings. The Elo system has also been used to predict the final league position of soccer teams [13]. By comparison relatively little work has been done on the dynamics of league tables themselves and the factors that influence their behaviour. Indeed, much of the work that has been done in this field originates in the USA to assess team performance in high scoring sports such as American football, baseball and basketball [14–18]–sports that exploit a conference system, rather than the traditional leagues preferred in soccer. So while much work has focused on US sports, less work has been published concerning soccer, and that which has been done has tended to focus on predicting match outcomes [19–25] and computing expected points totals [26–28] rather than focusing on the behaviour of the partial standings. Having said this, a few researchers have investigated the structure of soccer leagues. Notably, Lasek and Gagolewski [29] found that the traditional ‘*round-robin*’ league format, in which each team plays the other teams twice, once at home and once away, was not as efficacious at ranking teams as a two-stage competition. Shin et al. [30] comparing the dynamics of the English Premier (soccer) League with those of the National (American) Football League (NFL) in the USA, observed similarities between the two leagues despite the marked differences between the sports and the structures of the two competitions. As such, this suggests that the performance of soccer teams might be constrained by the structure of the league competitions in which they compete.

**Fig 1:**
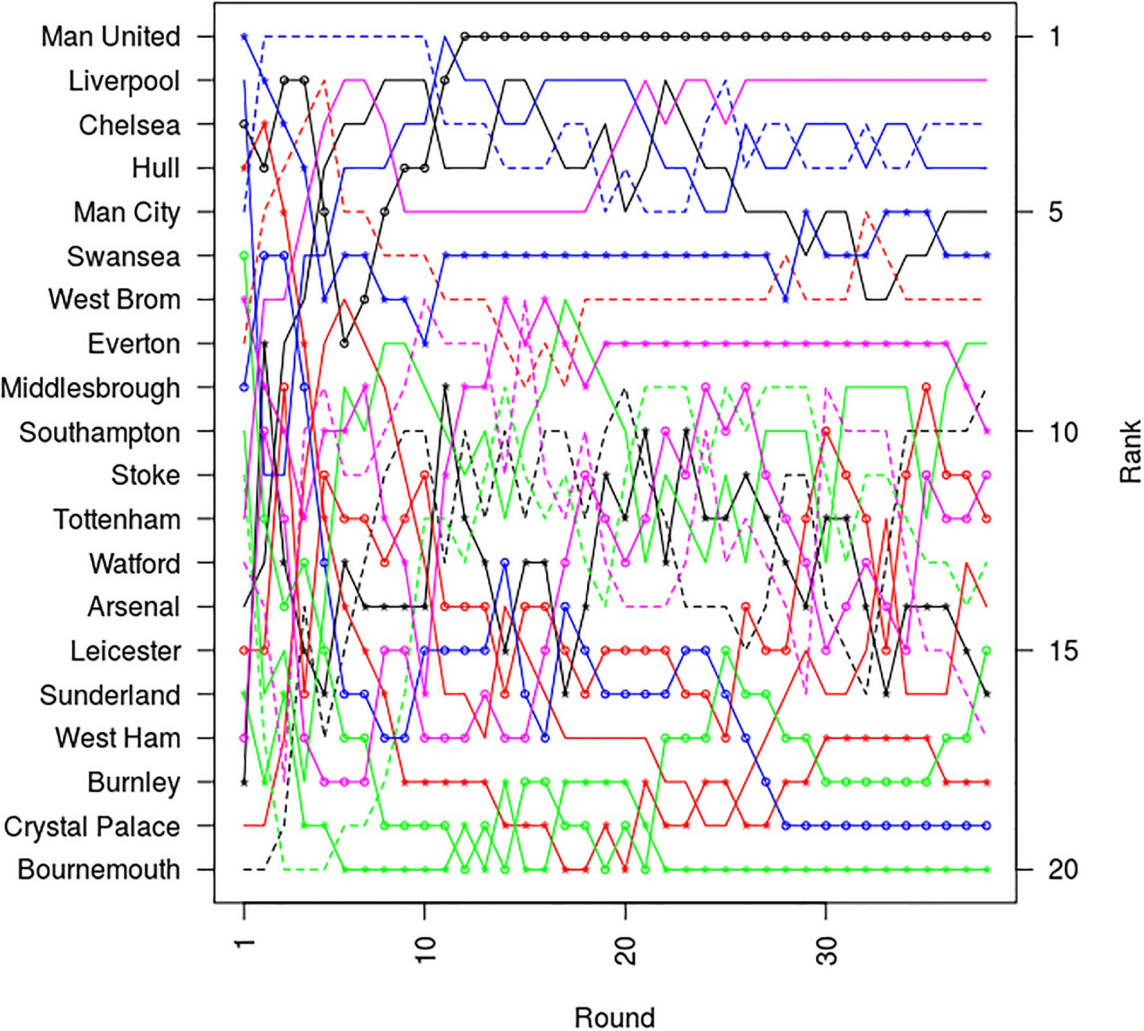
Change in team partial standings as the season progresses in the English Premier League for the 2016–17 season.

**Fig 2:**
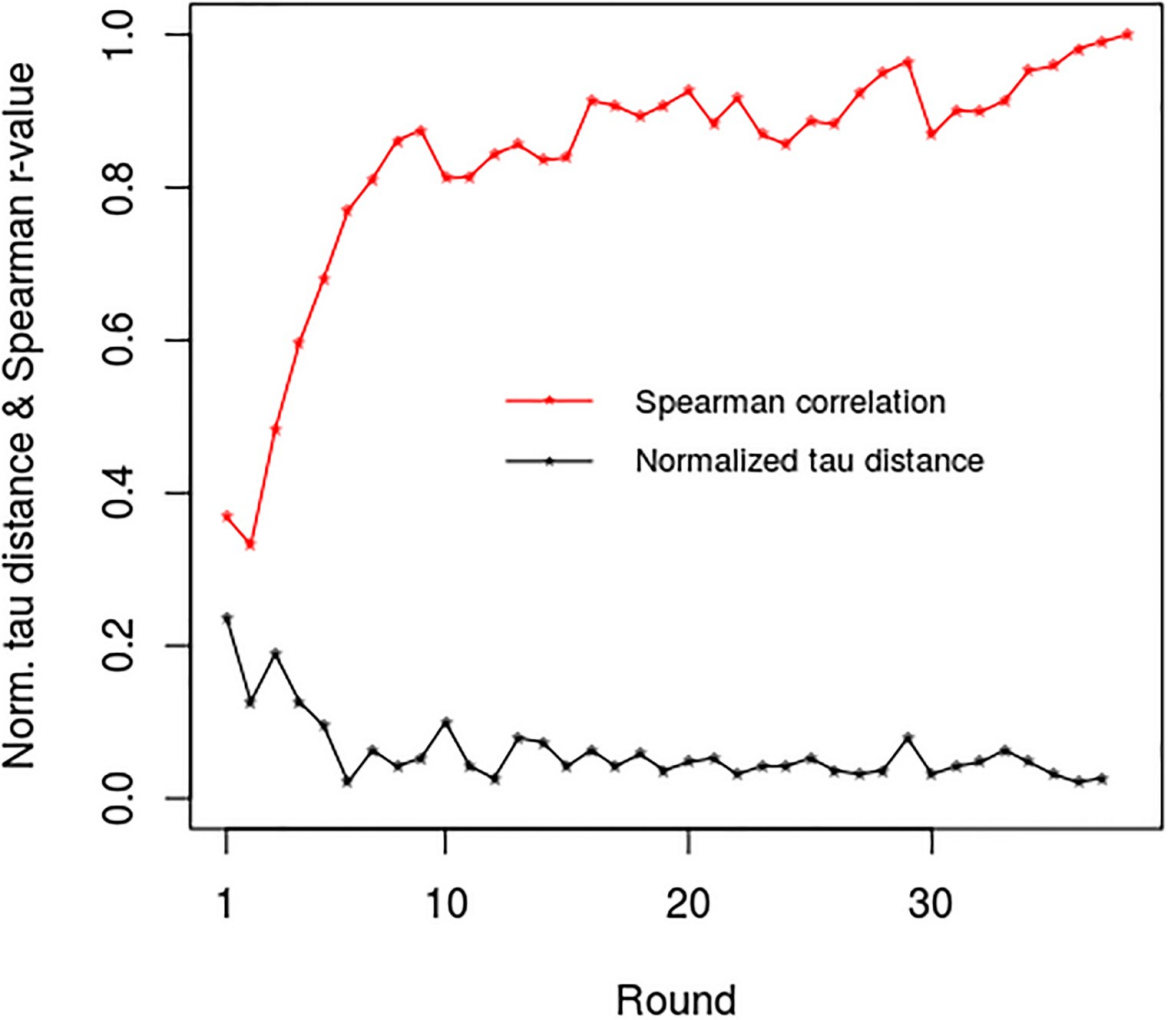
Normalized Kendall’s tau distances between partial standings for successive rounds of competition, together with Spearman rank correlations (r-values) between final standings and those for each round for season 2016–17 of the English Premier League.

In mathematical terms, soccer leagues can be viewed as a discrete state-space in which the competing teams change ranking state (i.e. standing position) as each round of competition is completed. So for example, the English Premier League, which consists of 20 teams who each play 38 games per season, can be viewed as a [20 × 38] matrix in which each team can occupy one of 20 unique states at the end of each round of competition. When viewed in this way it can be shown that leagues possess an inherent dynamic that constrains their behaviour, making it more difficult for teams to change league position as the season progresses [30]. This phenomenon is well illustrated in Fig 1, which shows how the standings of the teams in the English Premier League altered over the 38 rounds of competition during the 2016–17 season. From this, it can be seen that at the start of the season there was considerable crossing-over in partial standing position between the teams as they progressed from round to round. However as the competition progressed, the number of ‘cross-over’ events greatly diminishes, with only relatively few occurring after round 30. This is reflected in the normalized Kendall’s tau distance plot in Fig 2 for the same season, which shows the number of changes in ranking order between the standings for successive rounds of competition as the season progressed. This reveals that a rapid decrease in tau distance occurred at the start of the season, which subsequently slowed down as the season progressed, with little change occurring in the latter part of the season. If one also considers the Spearman rank correlations (r-values) between the team standings after each round of competition with the final standings at the end of the season (see Fig 2), it can be seen that the resulting correlation curve for season 2016–17 mirrors the tau distance curve, revealing that r-values >0.8 were achieved by round 10, with only relatively modest changes occurring after that. Given this, it may be that the partial standings themselves have potential as predictors of league outcome. However, there is a paucity of published work on the dynamics of partial standings in soccer leagues, with Shin et al.’s [30] study of the English Premier League being a notable exception. This study, although a valuable contribution, was nonetheless small, evaluating just the Premier League for seasons 2011–12 and 2012–13. As such, many unanswered questions remain concerning the general applicability of their findings. Consequently, there is a need for a more comprehensive study to evaluate whether or not the dynamics observed by Shin et al. are specific to the English Premier League or generally applicable to all soccer leagues. We therefore undertook the extensive study reported here, with the aim of better understanding the dynamics associated with partial standings in soccer leagues and assessing their potential for predicting league outcomes.

## Methods

The analysis utilized an ‘in-sample’ study design and was performed using publicly available historical match data (acquired from www.football-data.co.uk) for all four senior leagues in English soccer (Premier League, Championship, League 1, and League 2 –details listed in Table 1) for the 22 seasons from 1995–96 to 2016–17. 1995–96 was chosen as a cut-off point because this was the first season in which the Premier League contained just 20 teams–prior to this it comprised 22 teams. Analysis was performed using bespoke ‘in-house’ programs written in ‘R’ (version 3.0.2; open source statistical software), which incorporated an algorithm for computing league standings after each round of competition directly from match results [31]. In keeping with the system used in the English Premier League, teams were ranked firstly by total points, then by goal difference, and finally by goals scored, if the other two metrics were tied.

**Table 1:** Details of the four senior English soccer leagues.

League	Number of teams	Number of rounds	Number of matches	Type of competition
Premier League	20	38	380	Round-robin
Championship	24	46	552	Round-robin
League 1	24	46	552	Round-robin
League 2	24	46	552	Round-robin

For the respective leagues in each season the team standings after every round of competition (i.e. the partial standings) were computed. These were then used to compute: (i) the changes in the normalized Kendall’s tau distance (Eq 1) between the team standings in successive rounds of competition; and (ii) the changes in the Spearman r-value (Eq 2) between the team standings after each round of competition with the final standings at the end of the season. Kendall’s tau distance, which is a metric that counts the number of pair order disagreements between two ranking lists [32] was used because it directly reflects the number of changes in ranking order that occurred between the team standings in successive rounds of competition. In order to allow comparisons to be made between the various leagues, the tau distance was normalized to yield the fraction of discordant pairs as follows:

$$\tau =\frac{n_{d}}{n\left(n-1\right)/2}$$ (1)

where, *τ* is the normalized Kendall’s tau distance; *n* is the number of pairs of observations; and *n*_*d*_ is the number of discordant pairs.

In order to quantify the variance explained by the respective partial standings after each round of competition, the Spearman’s rank correlation coefficient was computed as follows:

$$r_{s}=1-\frac{6{\sum d}_{i}^{2}}{n\left(n^{2}-1\right)}$$ (2)

where, *r*_*s*_ is the Spearman’s rank correlation coefficient; and *d*_*i*_ is the difference between the two ranks for each pair of observations.

In order to describe the normalized tau distance and Spearman correlation curves for the respective leagues, several metrics were computed which captured various characteristics of the curves, such as the area under the curve (AUC), and the standardized AUC (i.e. AUC divided by the number of rounds of competition). Power law and logarithmic functions were also applied to the tau distance and Spearman correlation curves to establish the least-squares ‘best-fit’ curves for each league in the respective seasons. For the tau distance curves, the ‘best-fit’ conformed to the power law function:

$$\tau =b\times w^{a}$$ (3)

where; *τ* is the normalized tau distance; *w* is the round of competition; *a* is the power coefficient; and *b* is the multiplier coefficient. While the ‘best-fit’ for the Spearman correlation curves of the leagues approximated to a logarithmic function of the form:

$$r_{s}=d\times \text{ln}\left(w\right)+c$$ (4)

where; *r* is the Spearman r-value; *w* is the round of competition; *c* is the intercept coefficient; and *d* is the logarithmic coefficient.

For each season, coefficients a and b in the power law ‘best-fit’ function were determined by log transforming *τ* and *w*, and applying the ‘polyfit’ function in the ‘pracma’ package [33] in ‘R’. Coefficients c and d in the logarithmic function were computed using a linear model with *w* log transformed. Goodness-of-fit for the respective ‘best-fit’ curves was assessed using the R^2^ value.

In order to compare the dynamics of the four English leagues with a ‘baseline’, we constructed two hypothetical random leagues, one containing 20 teams and the other containing 24 teams. Computation of the respective ‘round-by-round’ partial standings for these random leagues was done by applying the same methodology as that outlined for the real leagues above [31], but using random home and away match scores instead of historical ones. For the 20-team random league, this involved generating 380 random home scores and 380 random away scores, both drawn from a Poisson distribution with a mean value of 1.325 (i.e. half of the average number of goals scored per match (mean = 2.650) for the Premier League over the 22-seasons), whereas for the 24-team league, 552 random home and away scores were generated from a Poisson distribution with a mean value of 1.291, which was half the 22-season average value of 2.582 goals per match for all the other leagues. We then attributed points (three points for a win, one for a draw, and zero points for a loss) to the random match results and computed the standings after each round of competition for both leagues. Importantly, in order to ensure that the behaviour of the random leagues was completely random and unbiased, no weighting was given for home advantage when generating the random results. In this way we could observe how the standings in a 20 or 24-team league would behave if the results were completely random. In order to simulate the behaviour of the random leagues over an equivalent number of seasons to the real leagues, this process was repeated 22 times and the resulting normalized tau distance and Spearman correlation curves acquired for each season. For the random leagues, the ‘best-fits’ for both the normalized tau distance and Spearman correlation curves corresponded to a power law function of the form shown in Eq 3.

To assess whether or not the order in which matches were played had any impact on the dynamic behaviour of the partial standings, *post-hoc* analysis was performed using Premier League data for season 2016–17. This involved computing the partial standings using the historical match results for this season, but with the order in which the matches were played changed. Specifically, we restructured the fixture list for Premier League for season 2016–17, so that the top 10 pre-season ranked teams played the teams ranked 11–20 during the first half of the season, with second half of the season reserved solely for fixtures in which the top 10 pre-season ranked teams played each other, and the lower ranked teams also played each other. In addition, we randomized the fixtures for season 2016–17, to create 22 separate ‘shuffled’ leagues. For all the real, restructured and shuffled leagues the normalized tau distances of the partial standings were computed, together with the respective ‘best-fit’ curves.

In addition to computing the partial standings, the end-of-season points totals for all the teams in the real and random leagues for the respective seasons were computed using the points allocation system outlined above. From this we were able to calculate the mean and standard deviation of the end-of-season points totals for each place in the final standings of the respective real and random leagues. The mean points difference between the adjacent places in the end-of-season standings was also computed for the real leagues.

Statistical analysis of the real and random leagues was performed using a two-tailed paired t-test applied to key metrics (coefficients *a*, *b*, *c* and *d* from the respective ‘best-fit’ models, AUC, and standardized AUC) derived from the individual tau distance and Spearman correlation curves for the respective seasons. The difference in the points totals for the teams in the real and randomized leagues was also assessed using a two-tailed paired t-test. For all tests, p values <0.05 were deemed to be significant.

In order to assess the predictive ‘power’ of the partial standings throughout the season we used the ‘round-by-round’ team standings computed for the respective real leagues for all 22 seasons from 1995–96 to 2016–17. Specifically, we used the partial standings at rounds 10, 20 and 30 and the final end-of-season standings to compute the respective transition probabilities. This was done by creating a separate adjacency matrix, *A*, with dimensions [20 × 20] for the Premier League and [24 × 24] for the other three leagues, for each of the three rounds (i.e. rounds 10, 20 and 30). For each respective round, matrix *A* contained the total number of transitions that occurred between the various partial and final standings over the 22 seasons, such that *A*_*i*,*j*_ represented the number of times a team transitioned from *j*^*th*^ place after either round 10, 20 or 30, to *i*^*th*^ place at the end of the season. The rows and columns of the adjacency matrix each summed to 22.

Having constructed matrix *A*, the transition probability matrix, *T*, was computed as follows:

$$T=A\times \left(\frac{1}{22}\right)$$ (5)

The rows and columns of the transition probability matrix both summed to one, with each element, *T*_*i*,*j*_, representing the historical probability of a team finishing the season in *i*^*th*^ place given a *j*^*th*^ place partial standing after round 10, 20 or 30.

## Results

**Fig 3:**
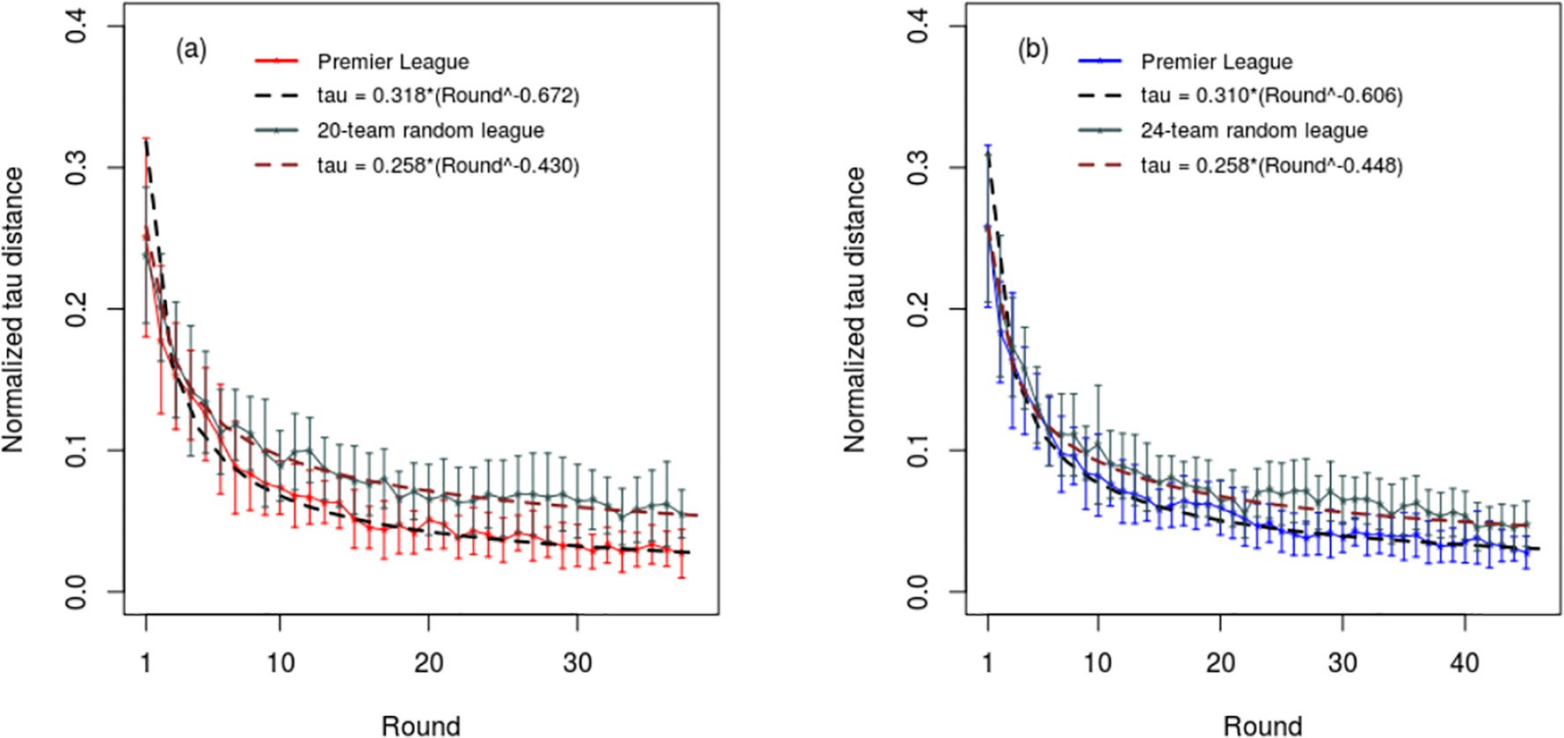
Normalized Kendall’s tau distance results after each round of competition for: (a) the Premier League, and (b) the Championship, together with results generated for a 20 team random league and a 24 team random league. The error bars represent one standard deviation. Best-fit power law curves are also shown.

Fig 3 shows the combined normalized tau distance results for the Premier League and Championship for seasons 1995–2017, together with the corresponding curves for the random 20-team and 24-team leagues. From this it can be seen that both the Premier League and Championship, together with the random leagues all have ‘best-fit’ curves of similar shape which conform to a power law. For the Premier League the correlation between the normalized tau distance and the power law curves for the respective seasons was R^2^ = 0.803 (SD = 0.079), whereas that for the: Championship was R^2^ = 0.838 (SD = 0.066); League 1 was R^2^ = 0.833 (SD = 0.089); and League 2 was R^2^ = 0.816 (SD = 0.080).

Interestingly, while the normalized tau distance curves for the real and random leagues both conform to a power law, those for the real league declined more rapidly than those for the random leagues, indicating that in the real leagues the standings tend to become ‘fixed in position’ relatively early in the season, with less ‘crossing-over’ in the team position occurring than would otherwise be the case if the results were completely random. With the exception of coefficient *b*, which did not reach significance for League 2, the differences between the real and corresponding random leagues were significant (p<0.05) for all the recorded tau distance metrics (i.e. AUC, standardized AUC, coefficient *a*, and coefficient *b*).

**Fig 4:**
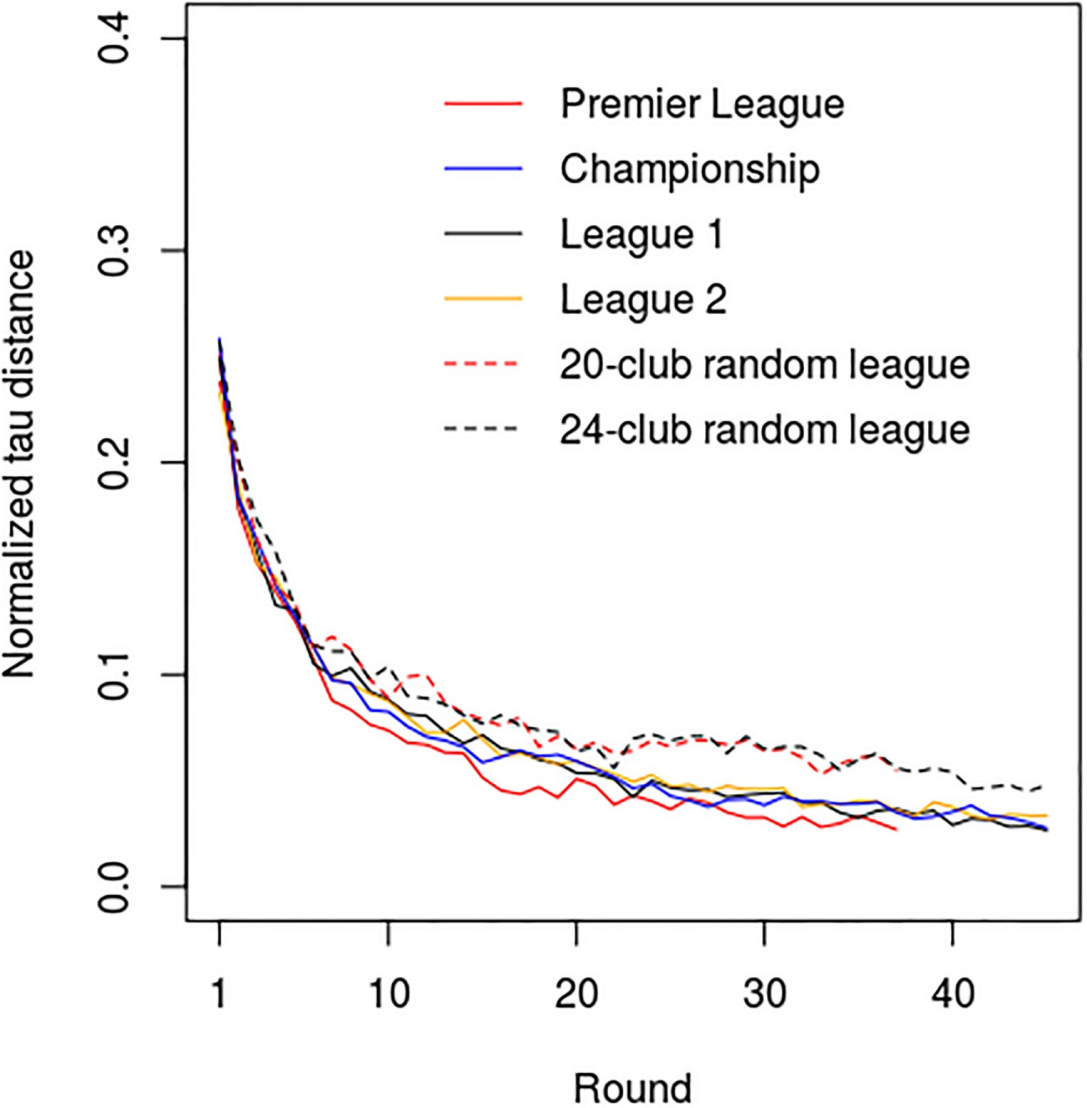
Mean ensemble normalized Kendall’s tau distance curves for the Premier League, Championship, League 1 and League 2 (seasons 1995–2017), together with results generated for the 20-team and 24-team random leagues (22 random seasons).

The comprehensive normalized tau distance results for all four real leagues and the two random leagues are presented in Fig 4 and Tables 2 and 3. These reveal that all the real 24-team leagues produced tau distance curves that were almost identical, with no significant differences occurring between any of the key metrics. Likewise, the tau distance metrics for the 20-team Premier League were similar to those of the other English leagues, with the only major difference being that the AUC metric was significantly lower for the Premier League (p<0.001), reflecting the fact that this league has only 38 rounds of competition, compared with 46 rounds for the other leagues. However, when the AUC was standardized, this difference disappeared. The only other significant difference between Premier League and the other leagues related to coefficient *a*, the absolute magnitude of which was slightly greater for the Premier League compared with that for the Championship (p = 0.046) and League 2 (p = 0.004). Collectively, these findings suggest that with respect to the normalized tau distance dynamics all the English 24-team leagues behaved in a very similar manner, with the Premier League also similar but with a tau distance curve that has a slightly different shape (Fig 4).

**Table 2:** Key parameters for the normalized tau distance curves for the respective leagues for seasons 1995–2017, together with coefficient and r-square values for the power law curve fits.

Parameter	PL mean (SD)	Champ mean (SD)	L1 mean (SD)	L2 mean (SD)	PL significance (p-values)	Champ. significance (p-values)	L1 significance (p-values)	L2 significance (p-values)
Coefficient a	-0.672 (0.124)	-0.606 (0.087)	-0.622 (0.087)	-0.574 (0.098)	0.046**, 0.192***, 0.004****	0.046*, 0.479***, 0.286****	0.192*, 0.479**, 0.110****	0.004*, 0.286**, 0.110***
Coefficient b	0.318 (0.087)	0.310 (0.057)	0.326 (0.066)	0.302 (0.077)	0.710**, 0.774***, 0.467****	0.710*, 0.322***, 0.692****	0.774*, 0.322**, 0.277****	0.467*, 0.692**, 0.277***
r-square	0.803 (0.079)	0.838 (0.066)	0.833 (0.089)	0.816 (0.080)	0.148**, 0.158***, 0.621****	0.148*, 0.816***, 0.395****	0.158*, 0.816**, 0.505****	0.621*, 0.395**, 0.505***
AUC	2.278 (0.290)	2.839 (0.330)	2.856 (0.312)	2.928 (0.291)	<0.001**, <0.001***, <0.001****	<0.001*, 0.843***, 0.319****	<0.001*, 0.843**, 0.378****	<0.001*, 0.319**, 0.378***
Standardized AUC	0.063 (0.008)	0.065 (0.008)	0.065 (0.007)	0.067 (0.007)	0.646**, 0.464***, 0.119****	0.646*, 0.842***, 0.321****	0.464*, 0.842**, 0.380****	0.119*, 0.321**, 0.380***

* p-value with respect to the Premier League;

** p-value with respect to the Championship;

*** p-value with respect to League 1;

**** p-value with respect to League 2.

PL–Premier League; Champ–Championship; L1 –League 1; L2 –League 2; Coefficient a–power coefficient; Coefficient b–multiplier coefficient; AUC–area under curve; SD–standard deviation.

**Table 3:** Key parameters for the normalized tau distance curves for the respective random leagues, together with coefficient and r-square values for the power law curve fits.

Parameter	20-team RL mean (SD)	24-team RL mean (SD)	Significance (p-values)
Coefficient a	-0.430 (0.135)	-0.448 (0.088)	0.502
Coefficient b	0.258 (0.070)	0.270 (0.056)	0.459
r-square	0.712 (0.184)	0.797 (0.077)	0.056
AUC	3.152 (0.408)	3.596 (0.413)	0.003
Standardized AUC	0.088 (0.011)	0.082 (0.009)	0.091

RL–random league; Coefficient a–power coefficient; Coefficient b–multiplier coefficient; AUC–area under curve; SD–standard deviation.

**Fig 5:**
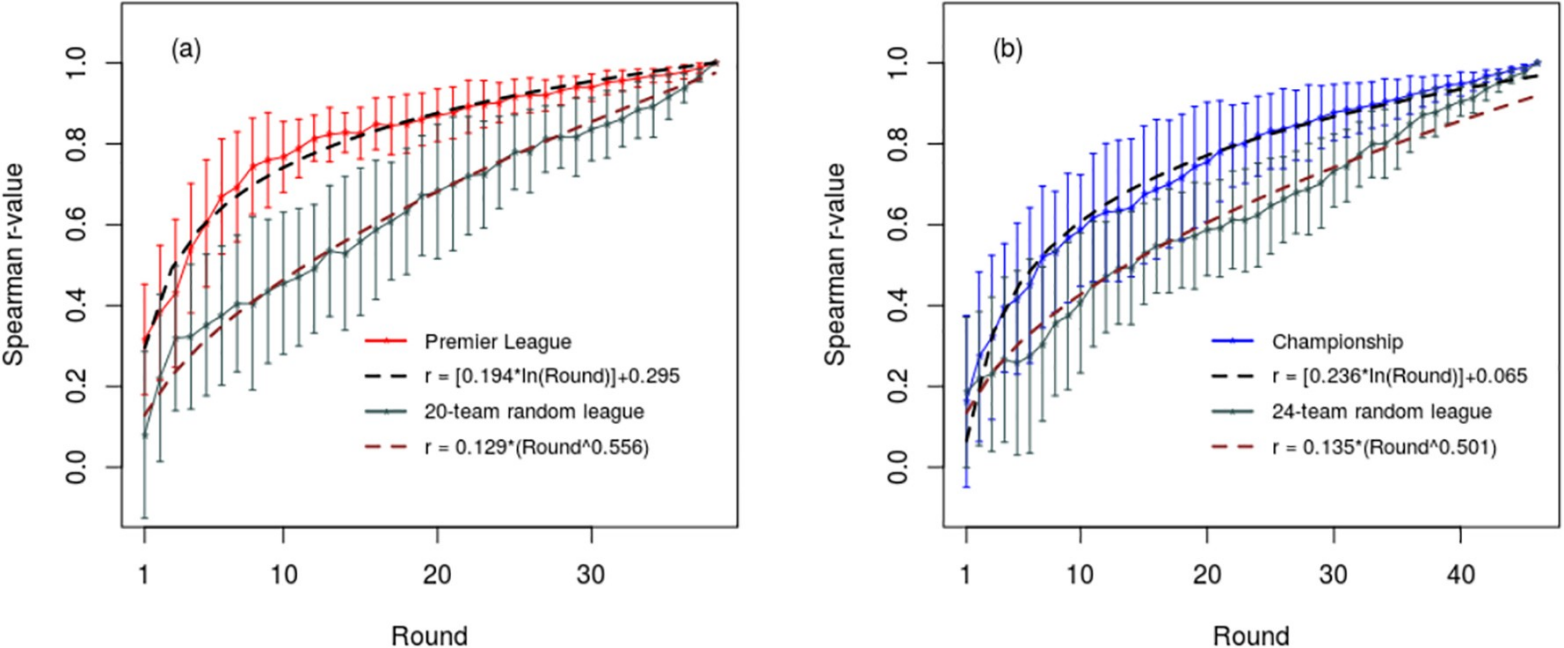
Spearman rank correlation (r-value) results after each round of competition for: (a) the Premier League, and (b) the Championship, together with results generated for a 20 team random league and a 24 team random league. The error bars represent one standard deviation. Best-fit logarithmic curves are also shown.

The Spearman correlation results for the real and random leagues shown in Fig 5 and Tables 4 and 5 reveal a similar picture to the tau distance results, with notable exception that the correlation curves for the real leagues approximated to a logarithmic function, whereas those of the random leagues conformed to a power law. For the Premier League the correlation between the Spearman r-value curves and the ‘best-fit’ logarithmic curves was R^2^ = 0.874 (SD = 0.058), whereas that for the: Championship was R^2^ = 0.899 (SD = 0.059); League 1 was R^2^ = 0.884 (SD = 0.058); and League 2 was R^2^ = 0.885 (SD = 0.082). Unlike the real leagues, the Spearman correlation curves of the random leagues conformed very closely to a power law, with *post-hoc* fitting of a power law function to the mean ensemble curve yielding R^2^ = 0.957 for the 20-team random league and R^2^ = 0.969 for the 24-team league. For all the real leagues the AUC was significantly greater (all p<0.001) than that for the corresponding random leagues. As such, this mirrors the tau distance results and indicates that for the real leagues the positions of the teams relative to their final standings, tends to become fixed earlier in the season than would otherwise be the case if the results were completely random.

**Table 4:** Key parameters for the Spearman correlation curves for the respective leagues for seasons 1995–2017, together with coefficient and r-square values for the logarithmic curve fits.

Parameter	PL mean (SD)	Champ. mean (SD)	L1 mean (SD)	L2 mean (SD)	PL significance (p-values)	Champ. significance (p-values)	L1 significance (p-values)	L2 significance (p-values)
Coefficient c	0.295 (0.156)	0.065 (0.234)	0.098 (0.274)	0.064 (0.208)	0.004**, 0.016***, <0.001****	0.004*, 0.623***, 0.977****	0.016*, 0.623**, 0.603****	<0.001*, 0.977**, 0.603***
Coefficient d	0.194 (0.043)	0.236 (0.053)	0.230 (0.076)	0.235 (0.060)	0.026**, 0.101***, 0.023****	0.026*, 0.699***, 0.947****	0.101*, 0.699**, 0.771****	0.023*, 0.947**, 0.771***
r-square	0.874 (0.058)	0.899 (0.059)	0.884 (0.058)	0.885 (0.082)	0.129**, 0.675***, 0.648****	0.129*, 0.403***, 0.596****	0.675*, 0.403**, 0.951****	0.648*, 0.596**, 0.951***
AUC	30.557 (1.796)	33.717 (4.091)	34.421 (2.790)	33.539 (2.215)	0.019**, <0.001***, <0.001****	0.019*, 0.548***, 0.963****	<0.001*, 0.548**, 0.219****	<0.001*, 0.963**, 0.219***
Standardized AUC	0.826 (0.049)	0.749 (0.093)	0.765 (0.062)	0.745 (0.049)	0.008**, <0.001***, <0.001****	0.008*, 0.548***, 0.963****	<0.001*, 0.548**, 0.219****	<0.001*, 0.963**, 0.219***

* p-value with respect to the Premier League;

** p-value with respect to the Championship;

*** p-value with respect to League 1;

**** p-value with respect to League 2.

PL–Premier League; Champ–Championship; L1 –League 1; L2 –League 2; Coefficient c–intercept coefficient; Coefficient d–logarithmic coefficient; AUC–area under curve; SD–standard deviation.

**Table 5:** Key parameters for the Spearman correlation curves for the respective random leagues, together with coefficient and r-square values for the mean ensemble power law curve fits.

Parameter	20-team RL mean (SD)	24-team RL mean (SD)	Significance (p-values)
Coefficient a	0.556*	0.501*	n.a.
Coefficient b	0.129*	0.135*	n.a.
r-square	0.957*	0.969*	n.a.
AUC: mean (SD)	23.649 (3.575)	28.170 (3.849)	<0.001
Standardized AUC: mean (SD)	0.639 (0.097)	0.626 (0.086)	0.626

RL–random league; Coefficient a–power coefficient; Coefficient b–multiplier coefficient; AUC–area under curve; SD–standard deviation

* Coefficients and r-square values computed *post hoc* using the mean ensemble Spearman correlation curves for the respective random leagues.

While little difference was observed in the Spearman correlation behaviour of the three 24-team leagues, noticeable differences were observed between the Premier League and the other leagues, with for example the intercept (coefficient *c*) and logarithmic (coefficient *d*) coefficients being larger (p = 0.004 and p = 0.026 respectively) in the Premier League compared to the Championship. This indicates that with the Premier League the position of the teams relative to their final standings tends to become fixed earlier in the season than in the other 24-team leagues, which all broadly behave in a similar manner.

**Fig 6:**
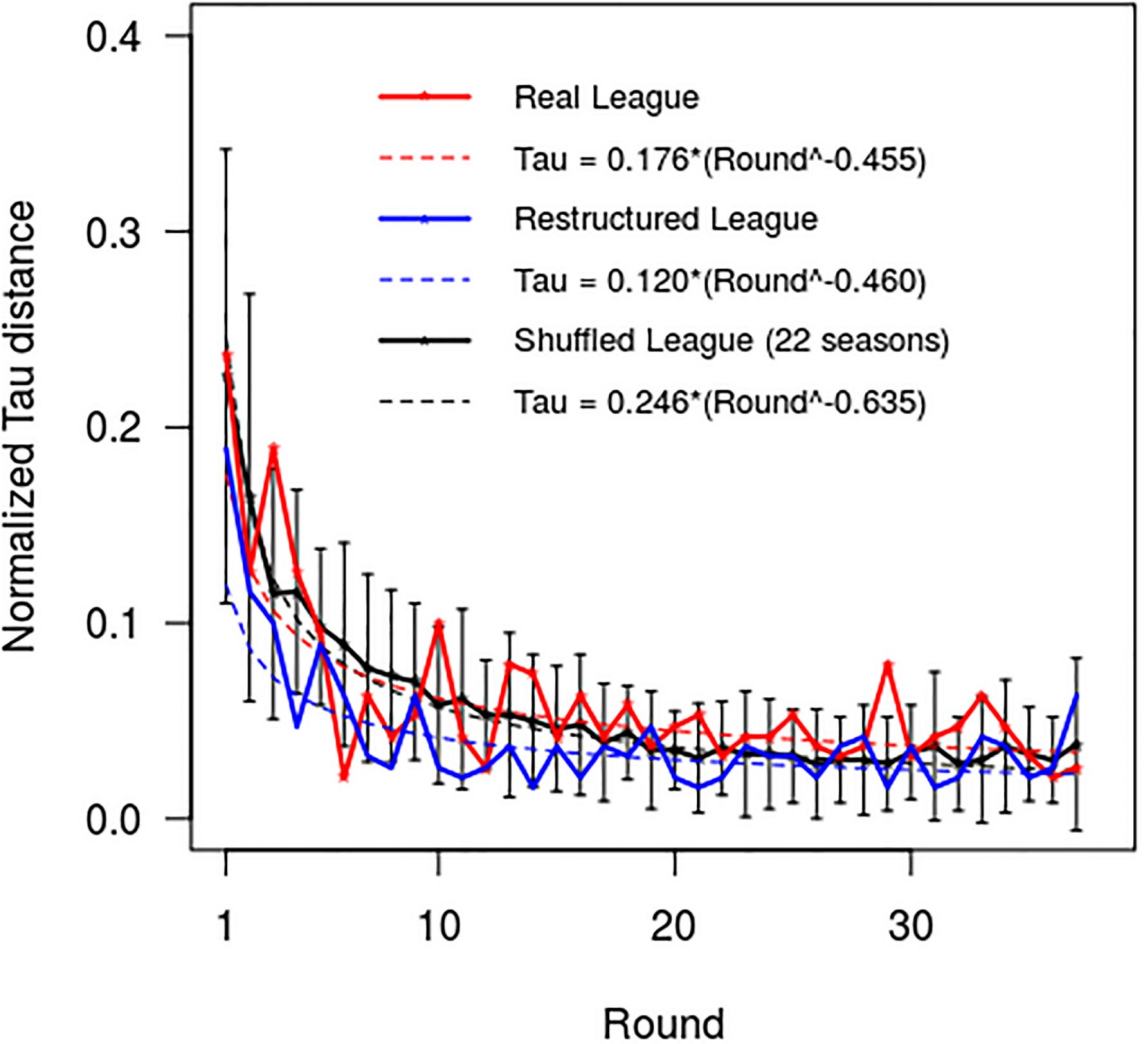
Normalized Kendall’s tau distances curves for the real, restructured and shuffled versions of the Premier League for season 2016–17. The error bars represent two standard deviations.

The results of the *post-hoc* analysis in which the fixtures for season 2016–17 were firstly restructured and then randomly shuffled are presented in Fig 6. These reveal that changing the order in which the matches were played, while keeping the match results unchanged, did indeed alter the behaviour of the partial standings of the Premier League. While all the normalized tau distance curves closely conformed to a power law (real league, R^2^ = 0.734; restructured league, R^2^ = 0.761, and shuffled leagues, R^2^ = 0.789 (SD = 0.116)), it was noticeable that reordering the games did change the shape of the ‘best-fit’ curves. For the real Premier League in season 2016–17 the best-fit curve had a power coefficient (coefficient *a*) = -0.455 and a multiplier coefficient (coefficient *b*) = 0.176, whereas for the restructured league the corresponding values were –0.460 and 0.120 respectively. The power and multiplier coefficients for the shuffled leagues, where -0.635 (SD = 0.082) and 0.246 (SD = 0.044) respectively.

**Fig 7:**
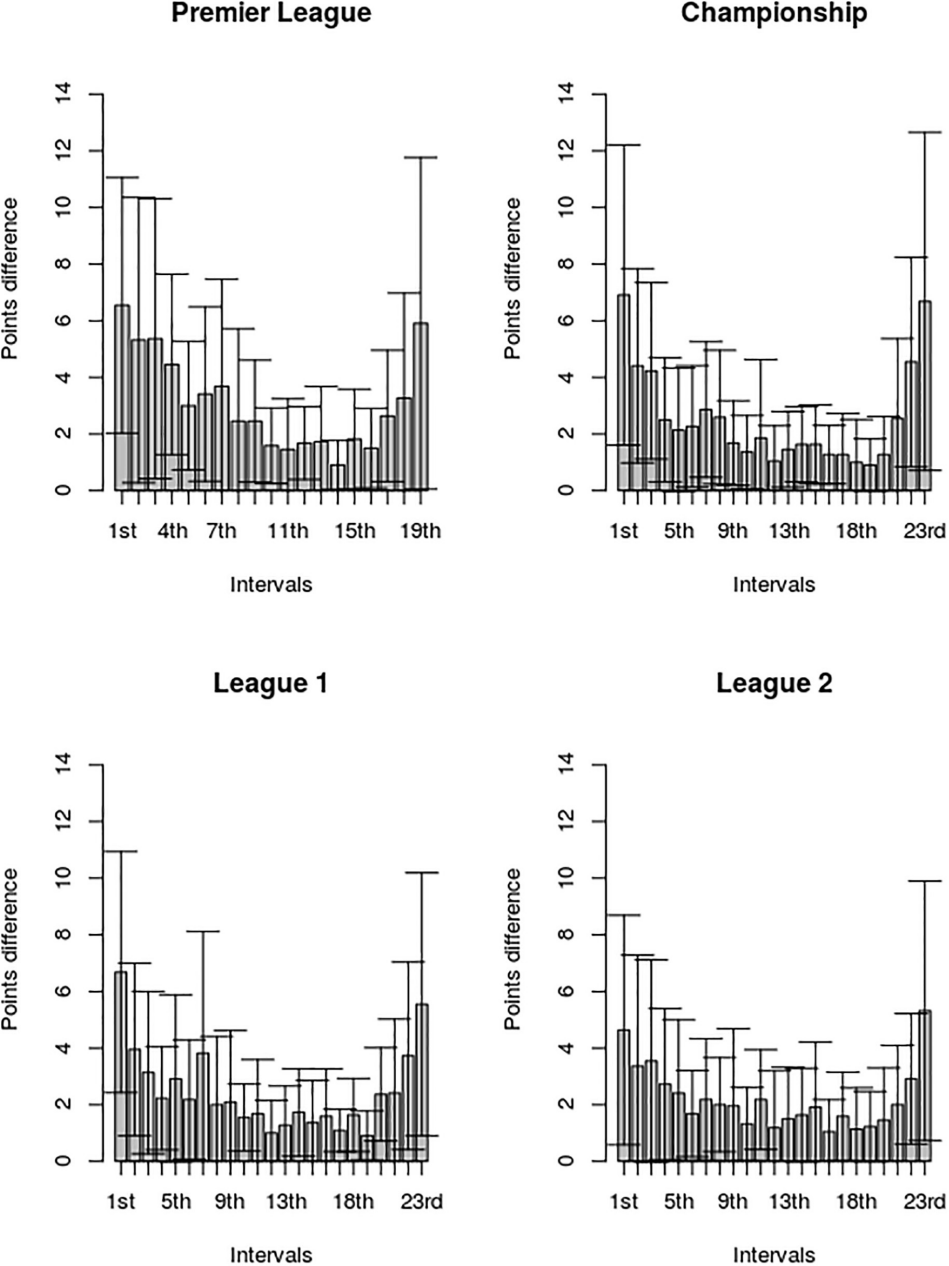
Mean difference in points between adjacent placings in the end-of-season standings for the Premier League, Championship, League 1 and League 2. The error bars represent one standard deviation.

The average points totals per place per season for the real and random leagues are presented in Table 6. From this it can be seen that the ‘total points’ distributions for the respective real leagues are markedly different from those for the corresponding random leagues. Noticeably, for each real league the top and bottom teams achieved much higher and lower points totals respectively than the teams with the same standings in the corresponding random leagues. As such, this indicates that in real life, points acquisition is not a random process, with the teams at the top of the leagues primarily prospering at the expense of the teams at the lower end of the leagues. This is supported by the results displayed in Fig 7, which show the mean points differences between each of the respective end of season standings. These reveal that for all four leagues, the distribution exhibits a slightly skewed ‘U’ shape, with the greatest points difference between the respective standings occurring at either end of the tables.

**Table 6:** Total points awarded per season for the Premier League, Championship, League 1 and League 2, together with respective random leagues.

Standing	PL	Champ.	L1	L2	20-team RL	24-team RL	Signif. 1	Signif. 2	Signif. 3	Signif. 4
	Mean (SD)	Mean (SD)	Mean (SD)	Mean (SD)	Mean (SD)	Mean (SD)	p-value	p-value	p-value	p-value
1^st^	85.8 (5.3)	94.0 (6.8)	93.5 (6.1)	89.7 (5.7)	66.6 (4.0)	79.9 (4.0)	<0.001	<0.001	<0.001	<0.001
2^nd^	79.3 (5.8)	87.0 (4.6)	86.8 (3.9)	85.1 (4.5)	64.1 (2.9)	75.9 (2.7)	<0.001	<0.001	<0.001	<0.001
3^rd^	74.0 (5.4)	82.6 (4.6)	82.8 (4.2)	81.7 (3.1)	61.3 (2.8)	74.0 (2.4)	<0.001	<0.001	<0.001	<0.001
4^th^	68.6 (4.7)	78.4 (3.9)	79.7 (3.9)	78.2 (3.7)	59.7 (2.6)	72.1 (1.9)	<0.001	<0.001	<0.001	<0.001
5^th^	64.1 (4.8	75.9 (3.0)	77.5 (4.4)	75.5 (3.6)	58.6 (2.3)	70.3 (1.9)	<0.001	<0.001	<0.001	<0.001
6^th^	61.1 (3.7)	73.8 (2.7)	74.5 (3.6)	73.0 (2.5)	57.4 (2.2)	69.2 (2.0)	0.001	<0.001	<0.001	<0.001
7^th^	57.7 (3.3)	71.5 (2.8)	72.4 (4.0)	71.4 (2.4)	56.4 (2.1)	68.3 (2.1)	0.140	<0.001	0.001	<0.001
8^th^	54.0 (3.9)	68.6 (3.4)	68.5 (2.2)	69.2 (2.9)	55.4 (1.8)	67.4 (1.9)	0.225	0.111	0.098	0.014
9^th^	51.6 (3.1)	66.0 (2.4)	66.5 (2.6)	67.2 (2.7)	54.2 (1.8)	66.1 (1.8)	0.009	0.861	0.504	0.140
10^th^	49.1 (2.4)	64.4 (2.8)	64.5 (2.9)	65.2 (2.7)	53.0 (1.7)	65.5 (1.7)	<0.001	0.069	0.151	0.766
11^th^	47.5 (2.4)	63.0 (2.4)	62.9 (2.8)	63.9 (2.7)	52.0 (2.0)	64.2 (1.1)	<0.001	0.043	0.024	0.633
12^th^	46.1 (2.3)	61.1 (2.7)	61.2 (2.7)	61.7 (2.8)	50.8 (2.1)	63.5 (1.2)	<0.001	0.002	0.001	0.015
13^th^	44.4 (2.4)	60.1 (2.4)	60.2 (2.5)	60.5 (2.5)	49.3 (2.3)	62.5 (1.3)	<0.001	0.001	<0.001	0.002
14^th^	42.7 (2.4)	58.6 (1.9)	59.0 (1.9)	59.0 (2.3)	47.7 (2.2)	61.8 (1.2)	<0.001	<0.001	<0.001	<0.001
15^th^	41.8 (2.2)	57.0 (1.9)	57.2 (1.9)	57.4 (2.3)	46.7 (2.0)	60.8 (1.2)	<0.001	<0.001	<0.001	<0.001
16^th^	40.0 (2.5)	55.4 (1.7)	55.9 (2.2)	55.5 (2.1)	45.7 (2.1)	59.7 (1.6)	<0.001	<0.001	<0.001	<0.001
17^th^	38.5 (2.5)	54.1 (1.9)	54.3 (1.6)	54.5 (2.2)	44.2 (2.8)	58.7 (1.5)	<0.001	<0.001	<0.001	<0.001
18^th^	35.8 (3.0)	52.8 (2.3)	53.2 (1.7)	52.9 (2.4)	42.4 (2.9)	57.3 (1.4)	<0.001	<0.001	<0.001	<0.001
19^th^	32.5 (3.3)	51.8 (2.6)	51.5 (1.4)	51.7 (1.8)	39.9 (3.0)	56.4 (1.7)	<0.001	<0.001	<0.001	<0.001
20^th^	26.6 (6.3)	50.9 (2.6)	50.6 (1.6)	50.5 (1.7)	37.1 (3.7)	55.4 (1.6)	<0.001	<0.001	<0.001	<0.001
21^st^	NA	49.6 (2.3)	48.3 (2.3)	49.0 (2.4)	NA	53.8 (2.0)	NA	<0.001	<0.001	<0.001
22^nd^	NA	47.1 (3.8)	45.9 (2.9)	47.0 (3.1)	NA	52.2 (1.8)	NA	<0.001	<0.001	<0.001
23^rd^	NA	42.5 (3.9)	42.1 (3.5)	44.1 (4.7)	NA	49.7 (2.6)	NA	<0.001	<0.001	<0.001
24^th^	NA	35.9 (6.1)	36.6 (5.7)	38.8 (6.8)	NA	46.5 (3.7)	NA	<0.001	<0.001	<0.001

PL–Premier League; Champ–Championship; L1 –League 1; L2 –League 2; RL–random league; Signif. 1—p-value with respect to the difference between points between the Premier League and the 20-team random league; Signif. 2—p-value with respect to the difference between points between the Championship and the 24-team random league; Signif. 3—p-value with respect to the difference between points between League 1 and the 24-team random league; Signif. 4—p-value with respect to the difference between points between League 2 and the 24-team random league.

The final standing transition probabilities for respective league positions at rounds 10, 20 and 30 of competition for the Premier League, Championship, League 1, and League 2 are presented in Tables 7–10. The probabilities presented in these tables are based on historical data (1995–2017) and show the likely final standing positions for all the league positions after rounds 10, 20 and 30. So for example, it can be seen that for the Premier League the team that is first at round 10 has a 40.9% chance of finishing the season in first place, an 18.2% chance of finishing in second place, and an 18.2% chance of finishing in third place. However, by round 30, the team in first place in the Premier League has a 72.7% chance of finishing first and an equal 13.6% chance of coming in second or third in the league. Correspondingly, at the other end of the league, the team in bottom position at round 10 has only a 27.3% chance of finishing last, whereas by round 30 this increases to 72.7%. As such, this confirms the findings of the tau distance and Spearman correlation analysis presented in Figs 3 and 5. Inspection of Tables 8–10 for the 24-team leagues reveals a similar picture to that for the Premier League, with the teams in the top positions increasingly likely to finish near the top of the league as the season progresses, and those near the bottom more likely to be relegated as the season progresses.

**Table 7:** Final standing transition probabilities for respective league positions at rounds 10, 20 and 30. Based on historical data (seasons 1995–2017) for the Premier League.

Round 10	EoS	EoS	EoS	EoS	EoS	EoS	EoS	EoS	EoS	EoS	EoS	EoS	EoS	EoS	EoS	EoS	EoS	EoS	EoS	EoS
Position	1st	2nd	3rd	4th	5th	6th	7th	8th	9th	10th	11th	12th	13th	14th	15th	16th	17th	18th	19th	20th
1st	0.41	0.18	0.18	0.09	0.05	0.05	0.00	0.05	0.00	0.00	0.00	0.00	0.00	0.00	0.00	0.00	0.00	0.00	0.00	0.00
2nd	0.14	0.41	0.18	0.05	0.09	0.05	0.05	0.05	0.00	0.00	0.00	0.00	0.00	0.00	0.00	0.00	0.00	0.00	0.00	0.00
3rd	0.18	0.23	0.09	0.14	0.14	0.00	0.09	0.05	0.05	0.05	0.00	0.00	0.00	0.00	0.00	0.00	0.00	0.00	0.00	0.00
4th	0.09	0.09	0.09	0.14	0.05	0.23	0.09	0.00	0.05	0.05	0.05	0.05	0.05	0.00	0.00	0.00	0.00	0.00	0.00	0.00
5th	0.14	0.05	0.14	0.14	0.09	0.05	0.05	0.05	0.09	0.09	0.00	0.05	0.05	0.00	0.00	0.00	0.05	0.00	0.00	0.00
6th	0.05	0.05	0.14	0.05	0.18	0.09	0.05	0.18	0.09	0.00	0.09	0.05	0.00	0.00	0.00	0.00	0.00	0.00	0.00	0.00
7th	0.00	0.00	0.05	0.14	0.09	0.09	0.05	0.05	0.09	0.00	0.05	0.05	0.09	0.09	0.09	0.05	0.05	0.00	0.00	0.00
8th	0.00	0.00	0.00	0.05	0.09	0.09	0.14	0.00	0.00	0.14	0.05	0.18	0.14	0.05	0.00	0.05	0.05	0.00	0.00	0.00
9th	0.00	0.00	0.05	0.05	0.05	0.14	0.14	0.14	0.05	0.14	0.09	0.00	0.00	0.00	0.00	0.05	0.00	0.05	0.09	0.00
10th	0.00	0.00	0.00	0.14	0.00	0.05	0.05	0.05	0.18	0.05	0.09	0.00	0.00	0.05	0.09	0.18	0.00	0.09	0.00	0.00
11th	0.00	0.00	0.09	0.00	0.09	0.05	0.05	0.14	0.05	0.05	0.05	0.05	0.09	0.18	0.00	0.09	0.05	0.00	0.00	0.00
12th	0.00	0.00	0.00	0.00	0.00	0.00	0.09	0.14	0.00	0.14	0.05	0.14	0.05	0.05	0.18	0.00	0.09	0.00	0.05	0.05
13th	0.00	0.00	0.00	0.05	0.05	0.05	0.05	0.05	0.05	0.09	0.05	0.09	0.14	0.09	0.05	0.09	0.05	0.09	0.00	0.00
14th	0.00	0.00	0.00	0.00	0.05	0.05	0.05	0.00	0.14	0.00	0.05	0.14	0.05	0.14	0.09	0.09	0.05	0.09	0.00	0.05
15th	0.00	0.00	0.00	0.00	0.00	0.05	0.05	0.00	0.09	0.05	0.00	0.09	0.09	0.14	0.00	0.14	0.00	0.09	0.14	0.09
16th	0.00	0.00	0.00	0.00	0.00	0.00	0.05	0.00	0.00	0.09	0.05	0.09	0.05	0.09	0.14	0.09	0.14	0.00	0.18	0.05
17th	0.00	0.00	0.00	0.00	0.00	0.00	0.00	0.00	0.05	0.09	0.09	0.00	0.14	0.00	0.09	0.14	0.09	0.18	0.09	0.05
18th	0.00	0.00	0.00	0.00	0.00	0.00	0.00	0.05	0.05	0.00	0.18	0.00	0.00	0.05	0.09	0.00	0.18	0.09	0.14	0.18
19th	0.00	0.00	0.00	0.00	0.00	0.00	0.00	0.00	0.00	0.00	0.05	0.05	0.05	0.05	0.09	0.00	0.09	0.23	0.14	0.27
20th	0.00	0.00	0.00	0.00	0.00	0.00	0.00	0.05	0.00	0.00	0.05	0.00	0.05	0.05	0.09	0.05	0.14	0.09	0.18	0.27
Round 20	EoS	EoS	EoS	EoS	EoS	EoS	EoS	EoS	EoS	EoS	EoS	EoS	EoS	EoS	EoS	EoS	EoS	EoS	EoS	EoS
Position	1st	2nd	3rd	4th	5th	6th	7th	8th	9th	10th	11th	12th	13th	14th	15th	16th	17th	18th	19th	20th
1st	0.55	0.23	0.09	0.09	0.00	0.05	0.00	0.00	0.00	0.00	0.00	0.00	0.00	0.00	0.00	0.00	0.00	0.00	0.00	0.00
2nd	0.23	0.36	0.23	0.14	0.00	0.05	0.00	0.00	0.00	0.00	0.00	0.00	0.00	0.00	0.00	0.00	0.00	0.00	0.00	0.00
3rd	0.14	0.18	0.23	0.27	0.05	0.05	0.05	0.05	0.00	0.00	0.00	0.00	0.00	0.00	0.00	0.00	0.00	0.00	0.00	0.00
4th	0.05	0.14	0.18	0.09	0.18	0.05	0.23	0.05	0.00	0.00	0.00	0.00	0.05	0.00	0.00	0.00	0.00	0.00	0.00	0.00
5th	0.00	0.09	0.09	0.27	0.36	0.00	0.05	0.00	0.09	0.05	0.00	0.00	0.00	0.00	0.00	0.00	0.00	0.00	0.00	0.00
6th	0.05	0.00	0.18	0.05	0.27	0.23	0.05	0.05	0.00	0.09	0.00	0.05	0.00	0.00	0.00	0.00	0.00	0.00	0.00	0.00
7th	0.00	0.00	0.00	0.05	0.14	0.09	0.18	0.09	0.05	0.09	0.14	0.05	0.00	0.09	0.05	0.00	0.00	0.00	0.00	0.00
8th	0.00	0.00	0.00	0.00	0.00	0.14	0.05	0.18	0.23	0.09	0.05	0.00	0.09	0.05	0.05	0.05	0.05	0.00	0.00	0.00
9th	0.00	0.00	0.00	0.00	0.00	0.05	0.18	0.27	0.05	0.05	0.00	0.18	0.14	0.00	0.05	0.05	0.00	0.00	0.00	0.00
10th	0.00	0.00	0.00	0.00	0.00	0.18	0.00	0.05	0.23	0.14	0.09	0.05	0.09	0.00	0.05	0.09	0.05	0.00	0.00	0.00
11th	0.00	0.00	0.00	0.00	0.00	0.00	0.18	0.05	0.14	0.05	0.14	0.14	0.18	0.00	0.05	0.00	0.05	0.00	0.05	0.00
12th	0.00	0.00	0.00	0.05	0.00	0.05	0.00	0.09	0.09	0.09	0.18	0.09	0.09	0.09	0.09	0.05	0.05	0.00	0.00	0.00
13th	0.00	0.00	0.00	0.00	0.00	0.09	0.05	0.05	0.05	0.14	0.09	0.09	0.05	0.05	0.05	0.14	0.05	0.05	0.05	0.05
14th	0.00	0.00	0.00	0.00	0.00	0.00	0.00	0.00	0.05	0.14	0.14	0.05	0.09	0.14	0.05	0.14	0.09	0.14	0.00	0.00
15th	0.00	0.00	0.00	0.00	0.00	0.00	0.00	0.00	0.05	0.05	0.05	0.14	0.00	0.09	0.18	0.18	0.05	0.14	0.09	0.00
16th	0.00	0.00	0.00	0.00	0.00	0.00	0.00	0.05	0.00	0.00	0.05	0.05	0.05	0.14	0.05	0.23	0.09	0.05	0.14	0.14
17th	0.00	0.00	0.00	0.00	0.00	0.00	0.00	0.00	0.00	0.00	0.05	0.09	0.05	0.09	0.14	0.05	0.14	0.23	0.14	0.05
18th	0.00	0.00	0.00	0.00	0.00	0.00	0.00	0.05	0.00	0.05	0.05	0.05	0.09	0.14	0.09	0.05	0.09	0.14	0.14	0.09
19th	0.00	0.00	0.00	0.00	0.00	0.00	0.00	0.00	0.00	0.00	0.00	0.00	0.05	0.05	0.14	0.00	0.18	0.18	0.32	0.09
20th	0.00	0.00	0.00	0.00	0.00	0.00	0.00	0.00	0.00	0.00	0.00	0.00	0.00	0.09	0.00	0.00	0.14	0.09	0.09	0.59
Round 30	EoS	EoS	EoS	EoS	EoS	EoS	EoS	EoS	EoS	EoS	EoS	EoS	EoS	EoS	EoS	EoS	EoS	EoS	EoS	EoS
Position	1st	2nd	3rd	4th	5th	6th	7th	8th	9th	10th	11th	12th	13th	14th	15th	16th	17th	18th	19th	20th
1st	0.73	0.14	0.14	0.00	0.00	0.00	0.00	0.00	0.00	0.00	0.00	0.00	0.00	0.00	0.00	0.00	0.00	0.00	0.00	0.00
2nd	0.18	0.55	0.18	0.09	0.00	0.00	0.00	0.00	0.00	0.00	0.00	0.00	0.00	0.00	0.00	0.00	0.00	0.00	0.00	0.00
3rd	0.09	0.23	0.50	0.14	0.05	0.00	0.00	0.00	0.00	0.00	0.00	0.00	0.00	0.00	0.00	0.00	0.00	0.00	0.00	0.00
4th	0.00	0.09	0.14	0.64	0.09	0.00	0.05	0.00	0.00	0.00	0.00	0.00	0.00	0.00	0.00	0.00	0.00	0.00	0.00	0.00
5th	0.00	0.00	0.00	0.14	0.46	0.23	0.18	0.00	0.00	0.00	0.00	0.00	0.00	0.00	0.00	0.00	0.00	0.00	0.00	0.00
6th	0.00	0.00	0.05	0.00	0.27	0.50	0.09	0.05	0.00	0.05	0.00	0.00	0.00	0.00	0.00	0.00	0.00	0.00	0.00	0.00
7th	0.00	0.00	0.00	0.00	0.14	0.23	0.27	0.18	0.09	0.00	0.09	0.00	0.00	0.00	0.00	0.00	0.00	0.00	0.00	0.00
8th	0.00	0.00	0.00	0.00	0.00	0.05	0.05	0.32	0.14	0.18	0.00	0.05	0.14	0.09	0.00	0.00	0.00	0.00	0.00	0.00
9th	0.00	0.00	0.00	0.00	0.00	0.00	0.18	0.14	0.32	0.09	0.18	0.05	0.00	0.00	0.00	0.00	0.05	0.00	0.00	0.00
10th	0.00	0.00	0.00	0.00	0.00	0.00	0.05	0.00	0.23	0.32	0.05	0.18	0.09	0.05	0.00	0.05	0.00	0.00	0.00	0.00
11th	0.00	0.00	0.00	0.00	0.00	0.00	0.05	0.09	0.05	0.14	0.23	0.18	0.09	0.09	0.09	0.00	0.00	0.00	0.00	0.00
12th	0.00	0.00	0.00	0.00	0.00	0.00	0.09	0.09	0.05	0.05	0.18	0.05	0.23	0.14	0.05	0.09	0.00	0.00	0.00	0.00
13th	0.00	0.00	0.00	0.00	0.00	0.00	0.00	0.14	0.05	0.00	0.14	0.23	0.14	0.05	0.05	0.14	0.05	0.05	0.00	0.00
14th	0.00	0.00	0.00	0.00	0.00	0.00	0.00	0.00	0.05	0.14	0.00	0.14	0.14	0.09	0.18	0.14	0.09	0.05	0.00	0.00
15th	0.00	0.00	0.00	0.00	0.00	0.00	0.00	0.00	0.05	0.00	0.05	0.09	0.05	0.09	0.14	0.27	0.05	0.14	0.09	0.00
16th	0.00	0.00	0.00	0.00	0.00	0.00	0.00	0.00	0.00	0.05	0.00	0.05	0.09	0.14	0.23	0.09	0.18	0.05	0.14	0.00
17th	0.00	0.00	0.00	0.00	0.00	0.00	0.00	0.00	0.00	0.00	0.09	0.00	0.05	0.14	0.14	0.09	0.14	0.23	0.09	0.05
18th	0.00	0.00	0.00	0.00	0.00	0.00	0.00	0.00	0.00	0.00	0.00	0.00	0.00	0.05	0.05	0.09	0.23	0.36	0.14	0.09
19th	0.00	0.00	0.00	0.00	0.00	0.00	0.00	0.00	0.00	0.00	0.00	0.00	0.00	0.05	0.09	0.00	0.18	0.14	0.41	0.14
20th	0.00	0.00	0.00	0.00	0.00	0.00	0.00	0.00	0.00	0.00	0.00	0.00	0.00	0.05	0.00	0.05	0.05	0.00	0.14	0.73

EoS–End of season

**Table 8:** Final standing transition probabilities for respective league positions at rounds 10, 20 and 30. Based on historical data (seasons 1995–2017) for the Championship.

Round 10	EoS	EoS	EoS	EoS	EoS	EoS	EoS	EoS	EoS	EoS	EoS	EoS	EoS	EoS	EoS	EoS	EoS	EoS	EoS	EoS	EoS	EoS	EoS	EoS
Position	1st	2nd	3rd	4th	5th	6th	7th	8th	9th	10th	11th	12th	13th	14th	15th	16th	17th	18th	19th	20th	21st	22nd	23rd	24th
1st	0.32	0.18	0.14	0.00	0.09	0.09	0.05	0.05	0.00	0.00	0.00	0.00	0.09	0.00	0.00	0.00	0.00	0.00	0.00	0.00	0.00	0.00	0.00	0.00
2nd	0.18	0.27	0.00	0.09	0.00	0.05	0.09	0.05	0.05	0.05	0.05	0.00	0.00	0.05	0.00	0.00	0.00	0.05	0.00	0.00	0.00	0.05	0.00	0.00
3rd	0.14	0.18	0.05	0.09	0.05	0.09	0.09	0.05	0.00	0.05	0.00	0.05	0.00	0.00	0.05	0.00	0.05	0.05	0.00	0.00	0.00	0.00	0.05	0.00
4th	0.05	0.05	0.18	0.09	0.09	0.00	0.09	0.05	0.05	0.09	0.14	0.05	0.00	0.05	0.05	0.00	0.00	0.00	0.00	0.00	0.00	0.00	0.00	0.00
5th	0.14	0.05	0.14	0.14	0.05	0.14	0.05	0.00	0.00	0.18	0.00	0.05	0.05	0.00	0.00	0.00	0.00	0.00	0.00	0.00	0.05	0.00	0.00	0.00
6th	0.05	0.00	0.05	0.09	0.00	0.09	0.00	0.23	0.05	0.00	0.00	0.05	0.09	0.00	0.00	0.00	0.14	0.05	0.05	0.09	0.00	0.00	0.00	0.00
7th	0.00	0.00	0.00	0.05	0.09	0.14	0.18	0.00	0.00	0.00	0.09	0.05	0.09	0.09	0.05	0.00	0.09	0.00	0.09	0.00	0.00	0.00	0.00	0.00
8th	0.00	0.00	0.09	0.14	0.09	0.00	0.09	0.09	0.00	0.09	0.09	0.05	0.05	0.00	0.05	0.00	0.00	0.05	0.00	0.00	0.09	0.00	0.05	0.00
9th	0.00	0.00	0.05	0.00	0.18	0.00	0.05	0.05	0.09	0.14	0.00	0.00	0.14	0.05	0.00	0.05	0.09	0.05	0.00	0.00	0.09	0.00	0.00	0.00
10th	0.00	0.14	0.05	0.05	0.00	0.09	0.05	0.00	0.09	0.00	0.00	0.09	0.05	0.05	0.09	0.05	0.05	0.00	0.09	0.09	0.00	0.00	0.00	0.00
11th	0.00	0.05	0.00	0.05	0.05	0.05	0.00	0.09	0.09	0.05	0.09	0.05	0.05	0.14	0.09	0.09	0.00	0.00	0.05	0.00	0.00	0.00	0.00	0.05
12th	0.00	0.00	0.00	0.05	0.00	0.09	0.05	0.09	0.05	0.00	0.00	0.00	0.05	0.05	0.18	0.05	0.09	0.00	0.14	0.05	0.05	0.00	0.05	0.00
13th	0.00	0.00	0.00	0.05	0.09	0.05	0.00	0.00	0.05	0.14	0.09	0.09	0.05	0.00	0.00	0.05	0.00	0.09	0.05	0.09	0.05	0.05	0.00	0.05
14th	0.05	0.05	0.18	0.05	0.05	0.05	0.05	0.00	0.00	0.00	0.09	0.05	0.05	0.05	0.00	0.05	0.00	0.00	0.14	0.00	0.09	0.00	0.00	0.05
15th	0.09	0.00	0.00	0.00	0.05	0.05	0.00	0.09	0.05	0.05	0.05	0.09	0.05	0.00	0.05	0.00	0.14	0.09	0.00	0.05	0.05	0.05	0.05	0.00
16th	0.00	0.00	0.05	0.00	0.00	0.00	0.05	0.05	0.00	0.00	0.09	0.05	0.05	0.05	0.09	0.14	0.05	0.14	0.05	0.05	0.00	0.09	0.05	0.00
17th	0.00	0.00	0.05	0.05	0.00	0.00	0.05	0.05	0.09	0.00	0.05	0.00	0.09	0.00	0.00	0.09	0.05	0.05	0.00	0.09	0.09	0.05	0.05	0.14
18th	0.00	0.00	0.00	0.00	0.00	0.00	0.05	0.00	0.09	0.00	0.05	0.05	0.00	0.14	0.05	0.05	0.00	0.00	0.09	0.14	0.05	0.14	0.05	0.09
19th	0.00	0.00	0.00	0.00	0.05	0.05	0.00	0.00	0.05	0.00	0.05	0.09	0.05	0.09	0.00	0.00	0.05	0.09	0.14	0.09	0.09	0.05	0.09	0.00
20th	0.00	0.05	0.00	0.00	0.05	0.00	0.00	0.05	0.18	0.09	0.00	0.05	0.00	0.05	0.05	0.05	0.05	0.00	0.00	0.09	0.05	0.14	0.09	0.00
21st	0.00	0.00	0.00	0.05	0.00	0.00	0.05	0.05	0.00	0.00	0.00	0.09	0.00	0.05	0.14	0.05	0.00	0.09	0.00	0.05	0.05	0.09	0.18	0.09
22nd	0.00	0.00	0.00	0.00	0.05	0.00	0.00	0.00	0.00	0.05	0.00	0.00	0.00	0.00	0.00	0.09	0.14	0.05	0.00	0.05	0.05	0.27	0.09	0.18
23rd	0.00	0.00	0.00	0.00	0.00	0.00	0.00	0.00	0.00	0.05	0.05	0.05	0.00	0.09	0.00	0.14	0.00	0.14	0.05	0.09	0.09	0.00	0.09	0.18
24th	0.00	0.00	0.00	0.00	0.00	0.00	0.00	0.00	0.05	0.00	0.05	0.00	0.05	0.05	0.09	0.09	0.05	0.05	0.09	0.00	0.09	0.05	0.14	0.18
Round 20	EoS	EoS	EoS	EoS	EoS	EoS	EoS	EoS	EoS	EoS	EoS	EoS	EoS	EoS	EoS	EoS	EoS	EoS	EoS	EoS	EoS	EoS	EoS	EoS
Position	1st	2nd	3rd	4th	5th	6th	7th	8th	9th	10th	11th	12th	13th	14th	15th	16th	17th	18th	19th	20th	21st	22nd	23rd	24th
1st	0.46	0.23	0.05	0.05	0.00	0.05	0.09	0.05	0.00	0.00	0.00	0.00	0.00	0.00	0.05	0.00	0.00	0.00	0.00	0.00	0.00	0.00	0.00	0.00
2nd	0.14	0.32	0.14	0.05	0.09	0.14	0.00	0.05	0.00	0.00	0.05	0.00	0.05	0.00	0.00	0.00	0.00	0.00	0.00	0.00	0.00	0.00	0.00	0.00
3rd	0.23	0.09	0.18	0.18	0.05	0.09	0.09	0.00	0.05	0.00	0.00	0.00	0.00	0.00	0.00	0.00	0.00	0.00	0.00	0.00	0.00	0.05	0.00	0.00
4th	0.00	0.00	0.18	0.14	0.27	0.05	0.18	0.05	0.00	0.05	0.00	0.00	0.00	0.00	0.00	0.09	0.00	0.00	0.00	0.00	0.00	0.00	0.00	0.00
5th	0.05	0.18	0.05	0.05	0.09	0.09	0.09	0.09	0.05	0.00	0.05	0.00	0.05	0.05	0.05	0.00	0.05	0.05	0.00	0.00	0.00	0.00	0.00	0.00
6th	0.00	0.05	0.09	0.05	0.05	0.18	0.14	0.14	0.09	0.14	0.00	0.00	0.00	0.00	0.05	0.00	0.00	0.05	0.00	0.00	0.00	0.00	0.00	0.00
7th	0.05	0.00	0.09	0.05	0.09	0.09	0.09	0.09	0.09	0.05	0.14	0.00	0.00	0.00	0.00	0.00	0.00	0.05	0.09	0.05	0.00	0.00	0.00	0.00
8th	0.00	0.00	0.05	0.18	0.05	0.14	0.05	0.09	0.05	0.14	0.00	0.00	0.14	0.05	0.00	0.00	0.05	0.00	0.05	0.00	0.00	0.00	0.00	0.00
9th	0.00	0.09	0.09	0.09	0.00	0.00	0.00	0.14	0.00	0.14	0.18	0.00	0.00	0.00	0.00	0.05	0.09	0.00	0.00	0.05	0.05	0.05	0.00	0.00
10th	0.00	0.05	0.00	0.09	0.00	0.05	0.00	0.05	0.14	0.09	0.09	0.05	0.14	0.05	0.05	0.00	0.14	0.00	0.00	0.05	0.00	0.00	0.00	0.00
11th	0.00	0.00	0.00	0.05	0.00	0.00	0.09	0.14	0.09	0.05	0.05	0.05	0.14	0.05	0.14	0.00	0.05	0.05	0.00	0.00	0.09	0.00	0.00	0.00
12th	0.00	0.00	0.05	0.00	0.00	0.05	0.00	0.00	0.00	0.05	0.05	0.14	0.18	0.09	0.09	0.09	0.09	0.05	0.05	0.05	0.00	0.00	0.00	0.00
13th	0.05	0.00	0.00	0.05	0.05	0.00	0.05	0.05	0.14	0.00	0.00	0.05	0.09	0.14	0.05	0.05	0.05	0.05	0.00	0.00	0.14	0.00	0.05	0.00
14th	0.05	0.00	0.00	0.00	0.18	0.00	0.00	0.00	0.00	0.05	0.05	0.09	0.05	0.05	0.09	0.00	0.14	0.05	0.09	0.09	0.05	0.00	0.00	0.00
15th	0.00	0.00	0.00	0.00	0.05	0.05	0.05	0.05	0.14	0.05	0.00	0.09	0.05	0.09	0.05	0.09	0.00	0.05	0.09	0.05	0.09	0.00	0.00	0.00
16th	0.00	0.00	0.00	0.00	0.00	0.00	0.00	0.00	0.05	0.09	0.05	0.09	0.00	0.18	0.14	0.14	0.05	0.00	0.05	0.09	0.05	0.05	0.00	0.00
17th	0.00	0.00	0.00	0.00	0.00	0.00	0.00	0.00	0.00	0.09	0.14	0.14	0.00	0.00	0.05	0.18	0.09	0.14	0.00	0.05	0.05	0.05	0.05	0.00
18th	0.00	0.00	0.05	0.00	0.00	0.00	0.09	0.00	0.00	0.00	0.00	0.09	0.09	0.09	0.00	0.05	0.09	0.09	0.14	0.05	0.14	0.05	0.00	0.00
19th	0.00	0.00	0.00	0.00	0.00	0.05	0.00	0.00	0.09	0.05	0.05	0.09	0.00	0.00	0.18	0.00	0.05	0.05	0.09	0.05	0.00	0.00	0.18	0.09
20th	0.00	0.00	0.00	0.00	0.05	0.00	0.00	0.00	0.00	0.00	0.00	0.00	0.05	0.09	0.00	0.05	0.00	0.09	0.14	0.23	0.00	0.14	0.05	0.14
21st	0.00	0.00	0.00	0.00	0.00	0.00	0.00	0.05	0.00	0.00	0.05	0.05	0.00	0.00	0.00	0.09	0.00	0.18	0.05	0.09	0.14	0.23	0.00	0.09
22nd	0.00	0.00	0.00	0.00	0.00	0.00	0.00	0.00	0.05	0.00	0.00	0.05	0.00	0.00	0.05	0.14	0.00	0.00	0.05	0.09	0.14	0.14	0.23	0.09
23rd	0.00	0.00	0.00	0.00	0.00	0.00	0.00	0.00	0.00	0.00	0.05	0.05	0.00	0.00	0.00	0.00	0.09	0.05	0.14	0.00	0.09	0.09	0.23	0.23
24th	0.00	0.00	0.00	0.00	0.00	0.00	0.00	0.00	0.00	0.00	0.05	0.00	0.00	0.09	0.00	0.00	0.00	0.05	0.00	0.05	0.00	0.18	0.23	0.36
Round 30	EoS	EoS	EoS	EoS	EoS	EoS	EoS	EoS	EoS	EoS	EoS	EoS	EoS	EoS	EoS	EoS	EoS	EoS	EoS	EoS	EoS	EoS	EoS	EoS
Position	1st	2nd	3rd	4th	5th	6th	7th	8th	9th	10th	11th	12th	13th	14th	15th	16th	17th	18th	19th	20th	21st	22nd	23rd	24th
1st	0.68	0.09	0.14	0.09	0.00	0.00	0.00	0.00	0.00	0.00	0.00	0.00	0.00	0.00	0.00	0.00	0.00	0.00	0.00	0.00	0.00	0.00	0.00	0.00
2nd	0.09	0.55	0.14	0.09	0.05	0.09	0.00	0.00	0.00	0.00	0.00	0.00	0.00	0.00	0.00	0.00	0.00	0.00	0.00	0.00	0.00	0.00	0.00	0.00
3rd	0.09	0.18	0.36	0.14	0.00	0.09	0.09	0.05	0.00	0.00	0.00	0.00	0.00	0.00	0.00	0.00	0.00	0.00	0.00	0.00	0.00	0.00	0.00	0.00
4th	0.05	0.14	0.18	0.14	0.14	0.14	0.14	0.09	0.00	0.00	0.00	0.00	0.00	0.00	0.00	0.00	0.00	0.00	0.00	0.00	0.00	0.00	0.00	0.00
5th	0.05	0.05	0.00	0.32	0.09	0.14	0.14	0.05	0.05	0.00	0.09	0.00	0.05	0.00	0.00	0.00	0.00	0.00	0.00	0.00	0.00	0.00	0.00	0.00
6th	0.00	0.00	0.05	0.09	0.27	0.18	0.09	0.18	0.00	0.05	0.00	0.00	0.05	0.00	0.00	0.05	0.00	0.00	0.00	0.00	0.00	0.00	0.00	0.00
7th	0.00	0.00	0.05	0.05	0.18	0.09	0.09	0.18	0.00	0.14	0.18	0.00	0.00	0.05	0.00	0.00	0.00	0.00	0.00	0.00	0.00	0.00	0.00	0.00
8th	0.00	0.00	0.05	0.00	0.09	0.14	0.09	0.05	0.18	0.23	0.00	0.00	0.09	0.00	0.05	0.00	0.05	0.00	0.00	0.00	0.00	0.00	0.00	0.00
9th	0.05	0.00	0.05	0.05	0.05	0.09	0.09	0.09	0.18	0.05	0.23	0.05	0.05	0.00	0.00	0.00	0.00	0.00	0.00	0.00	0.00	0.00	0.00	0.00
10th	0.00	0.00	0.00	0.00	0.05	0.00	0.09	0.14	0.00	0.14	0.05	0.05	0.14	0.14	0.00	0.05	0.09	0.00	0.00	0.05	0.00	0.05	0.00	0.00
11th	0.00	0.00	0.00	0.00	0.00	0.05	0.14	0.09	0.14	0.09	0.05	0.09	0.05	0.14	0.05	0.00	0.00	0.05	0.00	0.09	0.00	0.00	0.00	0.00
12th	0.00	0.00	0.00	0.05	0.05	0.00	0.00	0.05	0.14	0.09	0.09	0.05	0.05	0.09	0.14	0.00	0.05	0.05	0.05	0.00	0.09	0.00	0.00	0.00
13th	0.00	0.00	0.00	0.00	0.05	0.00	0.00	0.00	0.05	0.00	0.05	0.27	0.18	0.00	0.05	0.09	0.09	0.09	0.05	0.00	0.05	0.00	0.00	0.00
14th	0.00	0.00	0.00	0.00	0.00	0.00	0.00	0.00	0.00	0.00	0.09	0.09	0.09	0.09	0.14	0.09	0.18	0.09	0.09	0.00	0.05	0.00	0.00	0.00
15th	0.00	0.00	0.00	0.00	0.00	0.00	0.00	0.00	0.14	0.09	0.00	0.09	0.09	0.00	0.14	0.14	0.05	0.05	0.05	0.09	0.05	0.05	0.00	0.00
16th	0.00	0.00	0.00	0.00	0.00	0.00	0.05	0.05	0.00	0.05	0.14	0.05	0.14	0.00	0.09	0.05	0.14	0.00	0.05	0.09	0.09	0.00	0.00	0.05
17th	0.00	0.00	0.00	0.00	0.00	0.00	0.00	0.00	0.05	0.05	0.00	0.05	0.00	0.09	0.14	0.18	0.05	0.05	0.14	0.09	0.05	0.05	0.00	0.05
18th	0.00	0.00	0.00	0.00	0.00	0.00	0.00	0.00	0.00	0.05	0.00	0.09	0.00	0.14	0.09	0.05	0.09	0.14	0.23	0.00	0.09	0.00	0.05	0.00
19th	0.00	0.00	0.00	0.00	0.00	0.00	0.00	0.00	0.00	0.00	0.00	0.00	0.00	0.05	0.09	0.09	0.00	0.23	0.09	0.27	0.09	0.09	0.00	0.00
20th	0.00	0.00	0.00	0.00	0.00	0.00	0.00	0.00	0.00	0.00	0.00	0.09	0.00	0.14	0.00	0.14	0.14	0.09	0.05	0.05	0.14	0.14	0.05	0.00
21st	0.00	0.00	0.00	0.00	0.00	0.00	0.00	0.00	0.00	0.00	0.05	0.00	0.00	0.05	0.00	0.05	0.00	0.00	0.09	0.18	0.18	0.14	0.14	0.14
22nd	0.00	0.00	0.00	0.00	0.00	0.00	0.00	0.00	0.05	0.00	0.00	0.05	0.05	0.05	0.05	0.05	0.00	0.14	0.09	0.00	0.14	0.18	0.09	0.09
23rd	0.00	0.00	0.00	0.00	0.00	0.00	0.00	0.00	0.05	0.00	0.00	0.00	0.00	0.00	0.00	0.00	0.05	0.00	0.05	0.05	0.00	0.00	0.64	0.18
24th	0.00	0.00	0.00	0.00	0.00	0.00	0.00	0.00	0.00	0.00	0.00	0.00	0.00	0.00	0.00	0.00	0.05	0.05	0.00	0.05	0.00	0.32	0.05	0.50

EoS–End of season

**Table 9:** Final standing transition probabilities for respective league positions at rounds 10, 20 and 30. Based on historical data (seasons 1995–2017) for League 1.

Round 10	EoS	EoS	EoS	EoS	EoS	EoS	EoS	EoS	EoS	EoS	EoS	EoS	EoS	EoS	EoS	EoS	EoS	EoS	EoS	EoS	EoS	EoS	EoS	EoS
Position	1st	2nd	3rd	4th	5th	6th	7th	8th	9th	10th	11th	12th	13th	14th	15th	16th	17th	18th	19th	20th	21st	22nd	23rd	24th
1st	0.36	0.14	0.09	0.14	0.00	0.09	0.09	0.05	0.00	0.00	0.05	0.00	0.00	0.00	0.00	0.00	0.00	0.00	0.00	0.00	0.00	0.00	0.00	0.00
2nd	0.09	0.00	0.14	0.14	0.09	0.18	0.00	0.05	0.09	0.00	0.05	0.00	0.00	0.00	0.05	0.05	0.00	0.05	0.05	0.00	0.00	0.00	0.00	0.00
3rd	0.05	0.09	0.00	0.27	0.05	0.18	0.05	0.00	0.09	0.05	0.09	0.00	0.05	0.05	0.00	0.00	0.00	0.00	0.00	0.00	0.00	0.00	0.00	0.00
4th	0.00	0.09	0.18	0.09	0.14	0.05	0.00	0.05	0.00	0.14	0.00	0.14	0.00	0.05	0.00	0.05	0.00	0.00	0.00	0.00	0.00	0.00	0.05	0.00
5th	0.18	0.05	0.00	0.00	0.09	0.05	0.09	0.05	0.09	0.05	0.00	0.09	0.05	0.05	0.05	0.00	0.09	0.00	0.00	0.00	0.05	0.00	0.00	0.00
6th	0.05	0.09	0.05	0.09	0.09	0.09	0.00	0.09	0.00	0.09	0.14	0.05	0.05	0.00	0.05	0.05	0.05	0.00	0.00	0.00	0.00	0.00	0.00	0.00
7th	0.05	0.09	0.05	0.00	0.09	0.05	0.14	0.14	0.05	0.00	0.05	0.09	0.00	0.05	0.00	0.09	0.00	0.05	0.05	0.00	0.00	0.00	0.00	0.00
8th	0.00	0.14	0.05	0.00	0.00	0.05	0.05	0.09	0.00	0.05	0.05	0.05	0.18	0.00	0.05	0.05	0.05	0.00	0.00	0.09	0.05	0.00	0.05	0.00
9th	0.09	0.00	0.14	0.00	0.14	0.05	0.00	0.05	0.05	0.05	0.05	0.00	0.09	0.00	0.05	0.05	0.05	0.00	0.00	0.00	0.00	0.09	0.05	0.05
10th	0.00	0.09	0.05	0.00	0.05	0.00	0.09	0.09	0.18	0.05	0.00	0.05	0.05	0.09	0.14	0.00	0.00	0.00	0.00	0.05	0.05	0.00	0.00	0.00
11th	0.05	0.05	0.05	0.00	0.09	0.09	0.00	0.00	0.00	0.05	0.05	0.18	0.05	0.00	0.05	0.05	0.05	0.09	0.00	0.00	0.09	0.00	0.00	0.05
12th	0.05	0.00	0.05	0.05	0.00	0.00	0.05	0.05	0.09	0.00	0.00	0.05	0.09	0.05	0.09	0.14	0.05	0.05	0.14	0.05	0.00	0.00	0.00	0.00
13th	0.00	0.00	0.00	0.05	0.05	0.05	0.05	0.00	0.09	0.14	0.09	0.09	0.00	0.05	0.00	0.05	0.05	0.05	0.05	0.00	0.09	0.09	0.00	0.00
14th	0.00	0.00	0.09	0.00	0.00	0.00	0.09	0.00	0.05	0.14	0.00	0.05	0.09	0.00	0.00	0.09	0.14	0.00	0.00	0.05	0.00	0.09	0.05	0.09
15th	0.00	0.09	0.05	0.05	0.00	0.00	0.00	0.00	0.09	0.05	0.05	0.09	0.05	0.00	0.00	0.05	0.09	0.05	0.09	0.05	0.09	0.05	0.05	0.00
16th	0.05	0.00	0.00	0.14	0.00	0.00	0.05	0.09	0.00	0.00	0.00	0.05	0.00	0.09	0.00	0.00	0.09	0.09	0.05	0.05	0.09	0.00	0.05	0.14
17th	0.00	0.00	0.00	0.00	0.05	0.09	0.05	0.09	0.00	0.05	0.05	0.00	0.00	0.05	0.09	0.05	0.05	0.09	0.18	0.09	0.00	0.00	0.05	0.00
18th	0.00	0.05	0.00	0.00	0.00	0.00	0.00	0.05	0.05	0.00	0.05	0.00	0.14	0.09	0.14	0.09	0.05	0.00	0.00	0.05	0.05	0.09	0.05	0.09
19th	0.00	0.00	0.00	0.00	0.00	0.00	0.09	0.05	0.05	0.05	0.00	0.00	0.00	0.18	0.00	0.05	0.09	0.05	0.09	0.09	0.05	0.05	0.09	0.05
20th	0.00	0.00	0.00	0.00	0.09	0.00	0.00	0.05	0.00	0.00	0.05	0.00	0.05	0.00	0.14	0.00	0.05	0.05	0.09	0.00	0.18	0.14	0.14	0.00
21st	0.00	0.05	0.00	0.00	0.00	0.00	0.00	0.00	0.00	0.05	0.09	0.05	0.05	0.14	0.00	0.00	0.00	0.05	0.18	0.14	0.05	0.05	0.05	0.09
22nd	0.00	0.00	0.05	0.00	0.00	0.00	0.14	0.00	0.05	0.00	0.05	0.00	0.05	0.09	0.05	0.09	0.00	0.18	0.00	0.05	0.05	0.09	0.00	0.09
23rd	0.00	0.00	0.00	0.00	0.00	0.00	0.00	0.00	0.00	0.00	0.09	0.00	0.00	0.00	0.09	0.05	0.05	0.05	0.00	0.14	0.05	0.14	0.23	0.14
24th	0.00	0.00	0.00	0.00	0.00	0.00	0.00	0.00	0.00	0.05	0.00	0.00	0.00	0.00	0.00	0.00	0.05	0.14	0.05	0.14	0.09	0.14	0.14	0.23
Round 20	EoS	EoS	EoS	EoS	EoS	EoS	EoS	EoS	EoS	EoS	EoS	EoS	EoS	EoS	EoS	EoS	EoS	EoS	EoS	EoS	EoS	EoS	EoS	EoS
Position	1st	2nd	3rd	4th	5th	6th	7th	8th	9th	10th	11th	12th	13th	14th	15th	16th	17th	18th	19th	20th	21st	22nd	23rd	24th
1st	0.46	0.18	0.09	0.09	0.00	0.05	0.00	0.05	0.00	0.00	0.05	0.00	0.00	0.05	0.00	0.00	0.00	0.00	0.00	0.00	0.00	0.00	0.00	0.00
2nd	0.32	0.18	0.00	0.23	0.14	0.05	0.00	0.05	0.00	0.00	0.00	0.00	0.05	0.00	0.00	0.00	0.00	0.00	0.00	0.00	0.00	0.00	0.00	0.00
3rd	0.09	0.23	0.32	0.09	0.09	0.00	0.00	0.09	0.00	0.00	0.00	0.00	0.00	0.00	0.05	0.00	0.00	0.05	0.00	0.00	0.00	0.00	0.00	0.00
4th	0.09	0.09	0.14	0.05	0.23	0.14	0.05	0.05	0.05	0.05	0.00	0.05	0.00	0.05	0.00	0.00	0.00	0.00	0.00	0.00	0.00	0.00	0.00	0.00
5th	0.05	0.09	0.23	0.09	0.09	0.14	0.09	0.00	0.09	0.00	0.05	0.00	0.00	0.00	0.00	0.05	0.05	0.00	0.00	0.00	0.00	0.00	0.00	0.00
6th	0.00	0.05	0.00	0.05	0.18	0.05	0.09	0.09	0.18	0.09	0.05	0.09	0.09	0.00	0.00	0.00	0.00	0.00	0.00	0.00	0.00	0.00	0.00	0.00
7th	0.00	0.05	0.00	0.14	0.00	0.05	0.14	0.09	0.14	0.00	0.09	0.00	0.00	0.05	0.00	0.09	0.00	0.05	0.05	0.00	0.09	0.00	0.00	0.00
8th	0.00	0.00	0.00	0.09	0.09	0.14	0.05	0.00	0.05	0.05	0.14	0.09	0.05	0.00	0.14	0.00	0.05	0.05	0.00	0.05	0.00	0.00	0.00	0.00
9th	0.00	0.00	0.09	0.05	0.09	0.05	0.14	0.09	0.09	0.14	0.00	0.09	0.00	0.00	0.00	0.05	0.09	0.05	0.00	0.00	0.00	0.00	0.00	0.00
10th	0.00	0.09	0.05	0.05	0.00	0.05	0.05	0.05	0.09	0.05	0.09	0.14	0.09	0.09	0.09	0.05	0.00	0.00	0.00	0.00	0.00	0.00	0.00	0.00
11th	0.00	0.05	0.05	0.05	0.00	0.14	0.05	0.09	0.00	0.14	0.05	0.05	0.14	0.09	0.09	0.00	0.05	0.00	0.00	0.00	0.00	0.00	0.00	0.00
12th	0.00	0.00	0.00	0.05	0.00	0.09	0.05	0.09	0.00	0.09	0.14	0.09	0.05	0.09	0.00	0.05	0.18	0.00	0.00	0.00	0.00	0.05	0.00	0.00
13th	0.00	0.00	0.05	0.00	0.05	0.00	0.05	0.14	0.05	0.18	0.05	0.00	0.05	0.05	0.09	0.05	0.00	0.05	0.05	0.00	0.05	0.09	0.00	0.00
14th	0.00	0.00	0.00	0.00	0.05	0.05	0.05	0.09	0.05	0.05	0.09	0.05	0.05	0.14	0.09	0.09	0.00	0.05	0.00	0.05	0.00	0.00	0.00	0.09
15th	0.00	0.00	0.00	0.00	0.00	0.00	0.09	0.00	0.05	0.00	0.05	0.05	0.09	0.05	0.00	0.05	0.09	0.00	0.09	0.09	0.18	0.00	0.14	0.00
16th	0.00	0.00	0.00	0.00	0.00	0.00	0.05	0.00	0.05	0.00	0.00	0.14	0.00	0.09	0.05	0.09	0.05	0.09	0.23	0.00	0.14	0.00	0.00	0.05
17th	0.00	0.00	0.00	0.00	0.00	0.00	0.05	0.00	0.00	0.00	0.00	0.00	0.18	0.14	0.05	0.18	0.00	0.14	0.00	0.14	0.09	0.00	0.05	0.00
18th	0.00	0.00	0.00	0.00	0.00	0.00	0.00	0.05	0.00	0.09	0.05	0.00	0.00	0.00	0.14	0.05	0.09	0.00	0.09	0.14	0.05	0.18	0.09	0.00
19th	0.00	0.00	0.00	0.00	0.00	0.00	0.05	0.00	0.09	0.00	0.00	0.18	0.09	0.05	0.14	0.09	0.05	0.09	0.00	0.09	0.05	0.05	0.00	0.00
20th	0.00	0.00	0.00	0.00	0.00	0.00	0.00	0.00	0.00	0.00	0.05	0.00	0.00	0.00	0.00	0.05	0.18	0.23	0.14	0.00	0.09	0.14	0.09	0.05
21st	0.00	0.00	0.00	0.00	0.00	0.00	0.00	0.00	0.00	0.09	0.05	0.00	0.05	0.05	0.05	0.05	0.05	0.09	0.18	0.05	0.00	0.05	0.18	0.09
22nd	0.00	0.00	0.00	0.00	0.00	0.00	0.00	0.00	0.00	0.00	0.05	0.00	0.00	0.00	0.00	0.05	0.05	0.00	0.00	0.18	0.05	0.27	0.23	0.14
23rd	0.00	0.00	0.00	0.00	0.00	0.00	0.00	0.00	0.00	0.00	0.00	0.00	0.00	0.00	0.00	0.00	0.05	0.00	0.18	0.09	0.23	0.09	0.09	0.27
24th	0.00	0.00	0.00	0.00	0.00	0.05	0.00	0.00	0.05	0.00	0.00	0.00	0.05	0.05	0.05	0.00	0.00	0.09	0.00	0.14	0.00	0.09	0.14	0.32
Round 30	EoS	EoS	EoS	EoS	EoS	EoS	EoS	EoS	EoS	EoS	EoS	EoS	EoS	EoS	EoS	EoS	EoS	EoS	EoS	EoS	EoS	EoS	EoS	EoS
Position	1st	2nd	3rd	4th	5th	6th	7th	8th	9th	10th	11th	12th	13th	14th	15th	16th	17th	18th	19th	20th	21st	22nd	23rd	24th
1st	0.73	0.18	0.00	0.05	0.00	0.00	0.00	0.00	0.00	0.00	0.05	0.00	0.00	0.00	0.00	0.00	0.00	0.00	0.00	0.00	0.00	0.00	0.00	0.00
2nd	0.18	0.32	0.23	0.05	0.05	0.09	0.05	0.00	0.05	0.00	0.00	0.00	0.00	0.00	0.00	0.00	0.00	0.00	0.00	0.00	0.00	0.00	0.00	0.00
3rd	0.00	0.23	0.23	0.18	0.14	0.18	0.00	0.05	0.00	0.00	0.00	0.00	0.00	0.00	0.00	0.00	0.00	0.00	0.00	0.00	0.00	0.00	0.00	0.00
4th	0.05	0.09	0.14	0.32	0.27	0.09	0.05	0.00	0.00	0.00	0.00	0.00	0.00	0.00	0.00	0.00	0.00	0.00	0.00	0.00	0.00	0.00	0.00	0.00
5th	0.05	0.05	0.23	0.05	0.32	0.14	0.09	0.05	0.00	0.05	0.00	0.00	0.00	0.00	0.00	0.00	0.00	0.00	0.00	0.00	0.00	0.00	0.00	0.00
6th	0.00	0.05	0.09	0.23	0.09	0.18	0.09	0.05	0.05	0.00	0.00	0.09	0.05	0.05	0.00	0.00	0.00	0.00	0.00	0.00	0.00	0.00	0.00	0.00
7th	0.00	0.00	0.09	0.05	0.05	0.05	0.09	0.14	0.18	0.00	0.05	0.00	0.09	0.09	0.09	0.05	0.00	0.00	0.00	0.00	0.00	0.00	0.00	0.00
8th	0.00	0.05	0.00	0.05	0.00	0.05	0.09	0.14	0.14	0.14	0.14	0.09	0.05	0.05	0.00	0.00	0.05	0.00	0.00	0.00	0.00	0.00	0.00	0.00
9th	0.00	0.05	0.00	0.00	0.05	0.05	0.18	0.05	0.18	0.09	0.09	0.09	0.09	0.00	0.00	0.00	0.05	0.05	0.00	0.00	0.00	0.00	0.00	0.00
10th	0.00	0.00	0.00	0.05	0.00	0.14	0.00	0.14	0.14	0.18	0.09	0.05	0.14	0.05	0.00	0.00	0.00	0.05	0.00	0.00	0.00	0.00	0.00	0.00
11th	0.00	0.00	0.00	0.00	0.05	0.00	0.09	0.18	0.09	0.18	0.18	0.09	0.09	0.00	0.05	0.00	0.00	0.00	0.00	0.00	0.00	0.00	0.00	0.00
12th	0.00	0.00	0.00	0.00	0.00	0.05	0.05	0.00	0.09	0.05	0.00	0.14	0.27	0.14	0.00	0.05	0.05	0.05	0.00	0.05	0.00	0.05	0.00	0.00
13th	0.00	0.00	0.00	0.00	0.00	0.00	0.05	0.05	0.00	0.05	0.05	0.14	0.05	0.23	0.14	0.14	0.05	0.09	0.00	0.00	0.00	0.00	0.00	0.00
14th	0.00	0.00	0.00	0.00	0.00	0.00	0.14	0.09	0.05	0.00	0.05	0.05	0.05	0.05	0.09	0.18	0.05	0.18	0.00	0.00	0.00	0.00	0.05	0.00
15th	0.00	0.00	0.00	0.00	0.00	0.00	0.00	0.09	0.00	0.18	0.05	0.05	0.00	0.14	0.14	0.05	0.05	0.05	0.14	0.05	0.00	0.05	0.00	0.00
16th	0.00	0.00	0.00	0.00	0.00	0.00	0.00	0.00	0.00	0.00	0.09	0.00	0.00	0.05	0.09	0.14	0.23	0.09	0.05	0.09	0.09	0.05	0.05	0.00
17th	0.00	0.00	0.00	0.00	0.00	0.00	0.00	0.00	0.00	0.05	0.05	0.09	0.09	0.05	0.14	0.14	0.00	0.14	0.05	0.09	0.05	0.05	0.05	0.00
18th	0.00	0.00	0.00	0.00	0.00	0.00	0.00	0.00	0.00	0.00	0.05	0.05	0.00	0.00	0.14	0.14	0.14	0.00	0.09	0.05	0.18	0.05	0.09	0.05
19th	0.00	0.00	0.00	0.00	0.00	0.00	0.00	0.00	0.05	0.00	0.05	0.00	0.00	0.14	0.05	0.09	0.05	0.09	0.09	0.32	0.05	0.00	0.05	0.00
20th	0.00	0.00	0.00	0.00	0.00	0.00	0.00	0.00	0.00	0.00	0.05	0.09	0.00	0.00	0.05	0.00	0.18	0.05	0.14	0.18	0.14	0.09	0.05	0.00
21st	0.00	0.00	0.00	0.00	0.00	0.00	0.00	0.00	0.00	0.05	0.00	0.00	0.05	0.00	0.05	0.00	0.05	0.09	0.09	0.05	0.23	0.18	0.09	0.09
22nd	0.00	0.00	0.00	0.00	0.00	0.00	0.05	0.00	0.00	0.00	0.00	0.00	0.00	0.00	0.00	0.05	0.05	0.05	0.14	0.09	0.14	0.18	0.14	0.14
23rd	0.00	0.00	0.00	0.00	0.00	0.00	0.00	0.00	0.00	0.00	0.00	0.00	0.00	0.00	0.00	0.00	0.05	0.05	0.18	0.05	0.09	0.23	0.32	0.05
24th	0.00	0.00	0.00	0.00	0.00	0.00	0.00	0.00	0.00	0.00	0.00	0.00	0.00	0.00	0.00	0.00	0.00	0.00	0.05	0.00	0.05	0.09	0.14	0.68

EoS–End of season

**Table 10:** Final standing transition probabilities for respective league positions at rounds 10, 20 and 30. Based on historical data (seasons 1995–2017) for League 2.

Round 10	EoS	EoS	EoS	EoS	EoS	EoS	EoS	EoS	EoS	EoS	EoS	EoS	EoS	EoS	EoS	EoS	EoS	EoS	EoS	EoS	EoS	EoS	EoS	EoS
Position	1st	2nd	3rd	4th	5th	6th	7th	8th	9th	10th	11th	12th	13th	14th	15th	16th	17th	18th	19th	20th	21st	22nd	23rd	24th
1st	0.27	0.27	0.09	0.09	0.00	0.05	0.00	0.00	0.00	0.09	0.05	0.00	0.05	0.00	0.05	0.00	0.00	0.00	0.00	0.00	0.00	0.00	0.00	0.00
2nd	0.14	0.14	0.14	0.14	0.09	0.05	0.00	0.05	0.14	0.05	0.05	0.05	0.00	0.00	0.00	0.00	0.00	0.00	0.00	0.00	0.00	0.00	0.00	0.00
3rd	0.00	0.05	0.18	0.05	0.18	0.05	0.05	0.00	0.14	0.00	0.00	0.05	0.05	0.05	0.00	0.05	0.05	0.00	0.00	0.00	0.05	0.05	0.00	0.00
4th	0.14	0.00	0.18	0.14	0.09	0.05	0.00	0.05	0.05	0.14	0.00	0.00	0.05	0.00	0.09	0.00	0.00	0.00	0.05	0.00	0.00	0.00	0.00	0.00
5th	0.05	0.14	0.00	0.09	0.00	0.05	0.00	0.09	0.05	0.00	0.14	0.14	0.05	0.00	0.05	0.09	0.00	0.00	0.00	0.05	0.05	0.00	0.00	0.00
6th	0.09	0.05	0.00	0.05	0.23	0.05	0.00	0.05	0.05	0.09	0.09	0.05	0.05	0.05	0.05	0.05	0.00	0.00	0.05	0.00	0.00	0.00	0.00	0.00
7th	0.09	0.14	0.09	0.05	0.00	0.09	0.00	0.00	0.09	0.00	0.05	0.14	0.00	0.09	0.05	0.09	0.00	0.00	0.00	0.00	0.00	0.00	0.00	0.05
8th	0.05	0.05	0.14	0.00	0.00	0.09	0.14	0.05	0.00	0.14	0.05	0.00	0.00	0.00	0.00	0.05	0.05	0.00	0.00	0.00	0.14	0.00	0.05	0.05
9th	0.05	0.09	0.00	0.00	0.00	0.05	0.14	0.05	0.00	0.00	0.05	0.05	0.00	0.14	0.05	0.05	0.09	0.18	0.00	0.00	0.00	0.00	0.05	0.00
10th	0.09	0.00	0.00	0.09	0.05	0.00	0.05	0.14	0.09	0.05	0.05	0.05	0.05	0.00	0.00	0.05	0.00	0.05	0.09	0.09	0.00	0.00	0.00	0.05
11th	0.05	0.00	0.05	0.05	0.00	0.00	0.05	0.09	0.14	0.05	0.09	0.05	0.18	0.00	0.00	0.05	0.05	0.05	0.00	0.00	0.09	0.00	0.00	0.00
12th	0.00	0.05	0.05	0.09	0.00	0.05	0.05	0.00	0.00	0.05	0.00	0.00	0.05	0.00	0.05	0.00	0.09	0.14	0.05	0.09	0.09	0.09	0.05	0.00
13th	0.00	0.00	0.00	0.05	0.05	0.05	0.14	0.09	0.00	0.05	0.14	0.00	0.09	0.00	0.09	0.05	0.05	0.00	0.05	0.00	0.00	0.05	0.00	0.09
14th	0.00	0.05	0.05	0.05	0.14	0.09	0.09	0.09	0.00	0.05	0.00	0.05	0.05	0.00	0.05	0.05	0.09	0.05	0.00	0.00	0.00	0.09	0.00	0.00
15th	0.00	0.00	0.00	0.00	0.00	0.05	0.00	0.00	0.09	0.00	0.05	0.09	0.05	0.09	0.00	0.00	0.14	0.14	0.00	0.05	0.05	0.14	0.05	0.05
16th	0.00	0.00	0.00	0.00	0.05	0.05	0.14	0.09	0.05	0.05	0.00	0.09	0.09	0.18	0.09	0.00	0.05	0.00	0.00	0.09	0.00	0.00	0.00	0.00
17th	0.00	0.00	0.00	0.05	0.00	0.05	0.05	0.00	0.00	0.09	0.09	0.05	0.05	0.05	0.09	0.09	0.00	0.00	0.05	0.09	0.05	0.05	0.05	0.09
18th	0.00	0.00	0.00	0.00	0.00	0.14	0.00	0.09	0.00	0.05	0.00	0.14	0.00	0.05	0.09	0.05	0.00	0.09	0.09	0.00	0.05	0.00	0.09	0.09
19th	0.00	0.00	0.00	0.05	0.05	0.00	0.05	0.00	0.05	0.00	0.00	0.00	0.09	0.14	0.00	0.09	0.09	0.05	0.00	0.18	0.00	0.09	0.00	0.09
20th	0.00	0.00	0.00	0.00	0.00	0.00	0.05	0.00	0.09	0.05	0.09	0.05	0.05	0.00	0.05	0.05	0.00	0.05	0.14	0.05	0.09	0.00	0.05	0.18
21st	0.00	0.00	0.05	0.00	0.00	0.05	0.00	0.05	0.00	0.05	0.05	0.00	0.00	0.14	0.00	0.05	0.09	0.05	0.09	0.09	0.00	0.14	0.09	0.05
22nd	0.00	0.00	0.00	0.00	0.09	0.00	0.00	0.00	0.00	0.00	0.00	0.00	0.00	0.05	0.00	0.00	0.14	0.14	0.14	0.14	0.09	0.09	0.09	0.05
23rd	0.00	0.00	0.00	0.00	0.00	0.00	0.05	0.05	0.00	0.00	0.00	0.00	0.05	0.00	0.09	0.05	0.05	0.05	0.14	0.05	0.18	0.05	0.23	0.00
24th	0.00	0.00	0.00	0.00	0.00	0.00	0.00	0.00	0.00	0.00	0.00	0.00	0.00	0.00	0.09	0.09	0.00	0.00	0.09	0.05	0.09	0.18	0.23	0.18
Round 20	EoS	EoS	EoS	EoS	EoS	EoS	EoS	EoS	EoS	EoS	EoS	EoS	EoS	EoS	EoS	EoS	EoS	EoS	EoS	EoS	EoS	EoS	EoS	EoS
Position	1st	2nd	3rd	4th	5th	6th	7th	8th	9th	10th	11th	12th	13th	14th	15th	16th	17th	18th	19th	20th	21st	22nd	23rd	24th
1st	0.27	0.23	0.18	0.05	0.00	0.09	0.00	0.09	0.09	0.00	0.00	0.00	0.00	0.00	0.00	0.00	0.00	0.00	0.00	0.00	0.00	0.00	0.00	0.00
2nd	0.27	0.09	0.23	0.18	0.09	0.09	0.00	0.00	0.00	0.05	0.00	0.00	0.00	0.00	0.00	0.00	0.00	0.00	0.00	0.00	0.00	0.00	0.00	0.00
3rd	0.09	0.09	0.23	0.14	0.18	0.05	0.00	0.00	0.00	0.09	0.09	0.00	0.00	0.00	0.00	0.05	0.00	0.00	0.00	0.00	0.00	0.00	0.00	0.00
4th	0.18	0.27	0.09	0.09	0.05	0.00	0.00	0.09	0.05	0.00	0.05	0.05	0.00	0.05	0.05	0.00	0.00	0.00	0.00	0.00	0.00	0.00	0.00	0.00
5th	0.09	0.09	0.00	0.09	0.09	0.09	0.18	0.09	0.05	0.00	0.09	0.05	0.00	0.00	0.05	0.00	0.05	0.00	0.00	0.00	0.00	0.00	0.00	0.00
6th	0.05	0.00	0.05	0.00	0.05	0.14	0.14	0.00	0.32	0.09	0.00	0.00	0.05	0.05	0.05	0.00	0.00	0.00	0.00	0.00	0.00	0.05	0.00	0.00
7th	0.00	0.09	0.09	0.00	0.05	0.00	0.09	0.27	0.05	0.14	0.05	0.00	0.09	0.00	0.00	0.00	0.05	0.05	0.00	0.00	0.00	0.00	0.00	0.00
8th	0.05	0.05	0.09	0.05	0.05	0.00	0.05	0.05	0.09	0.09	0.05	0.09	0.05	0.05	0.09	0.00	0.00	0.05	0.05	0.00	0.00	0.00	0.00	0.05
9th	0.00	0.05	0.05	0.00	0.14	0.14	0.09	0.05	0.05	0.05	0.14	0.09	0.00	0.00	0.00	0.00	0.09	0.00	0.05	0.00	0.00	0.05	0.00	0.00
10th	0.00	0.05	0.00	0.09	0.05	0.05	0.05	0.05	0.14	0.09	0.09	0.05	0.05	0.00	0.00	0.14	0.05	0.00	0.00	0.05	0.05	0.00	0.00	0.00
11th	0.00	0.00	0.00	0.09	0.09	0.14	0.00	0.00	0.00	0.09	0.18	0.05	0.00	0.05	0.00	0.09	0.09	0.09	0.00	0.00	0.00	0.00	0.00	0.05
12th	0.00	0.00	0.00	0.14	0.05	0.00	0.14	0.00	0.05	0.05	0.00	0.09	0.14	0.14	0.05	0.05	0.00	0.05	0.05	0.00	0.05	0.00	0.00	0.00
13th	0.00	0.00	0.00	0.09	0.00	0.09	0.09	0.05	0.00	0.05	0.05	0.05	0.00	0.00	0.23	0.05	0.05	0.05	0.00	0.05	0.09	0.00	0.00	0.05
14th	0.00	0.00	0.00	0.00	0.05	0.05	0.00	0.05	0.00	0.00	0.05	0.09	0.00	0.09	0.00	0.14	0.14	0.00	0.05	0.14	0.05	0.09	0.05	0.00
15th	0.00	0.00	0.00	0.00	0.00	0.00	0.05	0.00	0.05	0.09	0.05	0.23	0.18	0.09	0.00	0.00	0.00	0.09	0.09	0.00	0.00	0.09	0.00	0.00
16th	0.00	0.00	0.00	0.00	0.00	0.00	0.05	0.18	0.00	0.09	0.00	0.05	0.05	0.05	0.05	0.00	0.09	0.09	0.14	0.00	0.05	0.09	0.00	0.05
17th	0.00	0.00	0.00	0.00	0.05	0.05	0.00	0.05	0.00	0.00	0.05	0.00	0.18	0.05	0.09	0.14	0.05	0.05	0.05	0.09	0.09	0.00	0.05	0.00
18th	0.00	0.00	0.00	0.00	0.00	0.00	0.05	0.00	0.05	0.00	0.05	0.05	0.14	0.09	0.05	0.00	0.05	0.23	0.09	0.09	0.05	0.00	0.05	0.00
19th	0.00	0.00	0.00	0.00	0.00	0.05	0.00	0.00	0.00	0.05	0.00	0.05	0.00	0.09	0.18	0.05	0.00	0.05	0.09	0.09	0.14	0.05	0.05	0.09
20th	0.00	0.00	0.00	0.00	0.05	0.00	0.05	0.00	0.00	0.00	0.05	0.05	0.00	0.18	0.05	0.14	0.14	0.00	0.05	0.09	0.05	0.05	0.09	0.00
21st	0.00	0.00	0.00	0.00	0.00	0.00	0.00	0.00	0.05	0.00	0.00	0.00	0.00	0.00	0.09	0.14	0.05	0.05	0.09	0.09	0.14	0.09	0.14	0.09
22nd	0.00	0.00	0.00	0.00	0.00	0.00	0.00	0.00	0.00	0.00	0.00	0.00	0.09	0.05	0.00	0.00	0.09	0.09	0.00	0.18	0.09	0.14	0.14	0.14
23rd	0.00	0.00	0.00	0.00	0.00	0.00	0.00	0.00	0.00	0.00	0.00	0.00	0.00	0.00	0.00	0.00	0.00	0.09	0.14	0.09	0.14	0.09	0.23	0.23
24th	0.00	0.00	0.00	0.00	0.00	0.00	0.00	0.00	0.00	0.00	0.00	0.00	0.00	0.00	0.00	0.05	0.05	0.00	0.09	0.05	0.05	0.23	0.23	0.27
Round 30	EoS	EoS	EoS	EoS	EoS	EoS	EoS	EoS	EoS	EoS	EoS	EoS	EoS	EoS	EoS	EoS	EoS	EoS	EoS	EoS	EoS	EoS	EoS	EoS
Position	1st	2nd	3rd	4th	5th	6th	7th	8th	9th	10th	11th	12th	13th	14th	15th	16th	17th	18th	19th	20th	21st	22nd	23rd	24th
1st	0.55	0.23	0.18	0.00	0.00	0.05	0.00	0.00	0.00	0.00	0.00	0.00	0.00	0.00	0.00	0.00	0.00	0.00	0.00	0.00	0.00	0.00	0.00	0.00
2nd	0.27	0.27	0.14	0.09	0.05	0.09	0.00	0.00	0.05	0.05	0.00	0.00	0.00	0.00	0.00	0.00	0.00	0.00	0.00	0.00	0.00	0.00	0.00	0.00
3rd	0.05	0.18	0.18	0.27	0.05	0.09	0.05	0.05	0.09	0.00	0.00	0.00	0.00	0.00	0.00	0.00	0.00	0.00	0.00	0.00	0.00	0.00	0.00	0.00
4th	0.05	0.05	0.05	0.32	0.27	0.00	0.00	0.14	0.09	0.05	0.00	0.00	0.00	0.00	0.00	0.00	0.00	0.00	0.00	0.00	0.00	0.00	0.00	0.00
5th	0.00	0.18	0.09	0.05	0.23	0.09	0.05	0.14	0.00	0.00	0.14	0.00	0.05	0.00	0.00	0.00	0.00	0.00	0.00	0.00	0.00	0.00	0.00	0.00
6th	0.05	0.09	0.23	0.00	0.09	0.14	0.09	0.18	0.00	0.05	0.05	0.05	0.00	0.00	0.00	0.00	0.00	0.00	0.00	0.00	0.00	0.00	0.00	0.00
7th	0.05	0.00	0.05	0.09	0.05	0.00	0.09	0.14	0.18	0.14	0.00	0.18	0.05	0.00	0.00	0.00	0.00	0.00	0.00	0.00	0.00	0.00	0.00	0.00
8th	0.00	0.00	0.00	0.00	0.09	0.14	0.23	0.05	0.14	0.05	0.05	0.09	0.00	0.05	0.05	0.00	0.05	0.00	0.00	0.05	0.00	0.00	0.00	0.00
9th	0.00	0.00	0.05	0.05	0.09	0.18	0.05	0.09	0.14	0.09	0.05	0.05	0.14	0.05	0.00	0.00	0.00	0.00	0.00	0.00	0.00	0.00	0.00	0.00
10th	0.00	0.00	0.05	0.09	0.00	0.18	0.00	0.09	0.00	0.09	0.18	0.00	0.05	0.09	0.05	0.00	0.05	0.05	0.00	0.00	0.05	0.00	0.00	0.00
11th	0.00	0.00	0.00	0.00	0.05	0.00	0.14	0.05	0.18	0.09	0.05	0.05	0.09	0.05	0.05	0.14	0.05	0.00	0.05	0.00	0.00	0.00	0.00	0.00
12th	0.00	0.00	0.00	0.00	0.00	0.00	0.23	0.00	0.00	0.14	0.23	0.00	0.00	0.05	0.05	0.14	0.05	0.05	0.00	0.00	0.05	0.00	0.00	0.05
13th	0.00	0.00	0.00	0.05	0.00	0.00	0.05	0.05	0.05	0.05	0.05	0.14	0.14	0.18	0.00	0.05	0.05	0.09	0.05	0.00	0.05	0.00	0.00	0.00
14th	0.00	0.00	0.00	0.00	0.00	0.00	0.00	0.05	0.05	0.05	0.09	0.14	0.05	0.14	0.18	0.09	0.09	0.05	0.05	0.00	0.00	0.00	0.00	0.00
15th	0.00	0.00	0.00	0.00	0.00	0.00	0.05	0.00	0.00	0.14	0.05	0.14	0.05	0.05	0.09	0.00	0.09	0.05	0.14	0.00	0.05	0.05	0.05	0.05
16th	0.00	0.00	0.00	0.00	0.00	0.05	0.00	0.00	0.00	0.00	0.00	0.05	0.09	0.05	0.23	0.09	0.09	0.14	0.00	0.05	0.05	0.09	0.05	0.00
17th	0.00	0.00	0.00	0.00	0.00	0.00	0.00	0.00	0.00	0.05	0.00	0.05	0.14	0.09	0.09	0.05	0.14	0.00	0.09	0.09	0.23	0.00	0.00	0.00
18th	0.00	0.00	0.00	0.00	0.00	0.00	0.00	0.00	0.00	0.00	0.05	0.05	0.05	0.09	0.14	0.09	0.14	0.05	0.05	0.09	0.09	0.14	0.00	0.00
19th	0.00	0.00	0.00	0.00	0.05	0.00	0.00	0.00	0.05	0.00	0.05	0.00	0.00	0.05	0.00	0.05	0.00	0.14	0.09	0.23	0.05	0.05	0.18	0.05
20th	0.00	0.00	0.00	0.00	0.00	0.00	0.00	0.00	0.00	0.00	0.00	0.00	0.09	0.05	0.05	0.18	0.05	0.05	0.09	0.05	0.09	0.09	0.09	0.14
21st	0.00	0.00	0.00	0.00	0.00	0.00	0.00	0.00	0.00	0.00	0.00	0.05	0.05	0.00	0.05	0.09	0.00	0.32	0.05	0.14	0.14	0.09	0.05	0.00
22nd	0.00	0.00	0.00	0.00	0.00	0.00	0.00	0.00	0.00	0.00	0.00	0.00	0.00	0.00	0.00	0.05	0.09	0.00	0.09	0.14	0.05	0.23	0.27	0.09
23rd	0.00	0.00	0.00	0.00	0.00	0.00	0.00	0.00	0.00	0.00	0.00	0.00	0.00	0.05	0.00	0.00	0.05	0.05	0.18	0.09	0.09	0.09	0.18	0.23
24th	0.00	0.00	0.00	0.00	0.00	0.00	0.00	0.00	0.00	0.00	0.00	0.00	0.00	0.00	0.00	0.00	0.05	0.00	0.09	0.09	0.05	0.18	0.14	0.41

EoS–End of season

## Discussion

From the analysis presented above it can be seen that a clear and consistent picture emerges, namely that all the English soccer leagues exhibit very similar behaviour with regard to the dynamics of the partial standings. For all leagues, the number of ‘cross-over’ events occurring, indicated by the change in the normalized tau distance between successive rounds of competition, rapidly decreases as the season progresses, irrespective of the actual teams involved in the competition. In all the leagues, this reduction in tau distance conforms closely to a power law (R^2^ >0.8). This indicates that as the season commences the best teams quickly rise to the top of their respective leagues, while the poorest teams equally quickly sink to the bottom, with the middle-ranking teams occupying the space in between. From this it can be surmised that being more consistent than their competitors, the best teams in each league quickly open up a ‘points gap’ at the top of the table, while those at the bottom tend to fall adrift during the same period. Consequently, ‘points gaps’ open up at both ends of the table, which become increasingly difficult to transcend as the season progresses. Indeed, such is the magnitude of these ‘points gaps’ compared with the points available in each round of competition (as illustrated in Fig 7), that it becomes increasingly more difficult for the teams to cross-over in the standings, with the result that tau distance dramatically reduces as the season progresses. When late season ‘cross-over’ events do occur, these tend to be mainly focused on mid-table where the point differential between the standings is smaller and thus easier to transcend, as illustrated in Fig 1.

If the Spearman correlation curves for the respective real leagues are compared with their random counterparts, it can be seen that the partial standings converge towards the end-of-season league positions much more quickly as the season progresses in the real leagues compared with the random leagues. For example, after ten rounds of competition in the Premier League, the mean Spearman r-value is 0.767, compared with just 0.455 for the equivalent random league. As such, this clearly indicates that the process is not random and that in real-life the tranche of teams that emerge at the top and bottom of soccer leagues early in the season consistently tend to perform better or worse than the other teams, maintaining ‘head starts’ that are difficult to overturn. This trend is well illustrated by the results in Tables 7–10. For example, from Table 7 it can be seen that the team in first place in the Premier League after round 10 has a 77.3% chance of finishing in the top three, while those in last place at the same point in the season have a 54.6% chance of being relegated. Indeed by round 10, the partial standings give a reasonable indication of final league position, with, in the case of the Premier League, 76.7% (i.e. R^2^ = 0.767) of the variance in the final standings being explained, even though it is still relatively early in the season. However, by round 20 the partial standings become a much better predictor of the end of season position, explaining 87.0% of the variance in the final Premier League standings—a figure that increases to 93.9% by round 30. Similar pictures emerge for the other leagues, with the Spearman r-values strongly conforming to a logarithmic function in all four English leagues (mean R^2^ >0.88). Collectively, these findings suggest that with respect to soccer leagues, the partial standings may have potential as a predictor of season outcome. While systems have been developed that use ranking algorithms [13] and expected points totals [7, 8] to predict end-of-season outcome, these tend to be complex and can be difficult to use. By comparison, it is relatively easy to use the transition probabilities computed from the partial standings (Tables 7–10) to give a ‘first approximation’ of likely end-of-season league position. As such, the partial standings appear to have potential as a simple easy-to-use tool for predicting final league position. Notwithstanding this, it is important to appreciate the limitations of this approach. The partial standings of teams are a poor predictor of the outcome of individual soccer matches, and more sophisticated methodologies are required in order to accurately predict match results [9, 19, 22, 24, 25, 34]. Therefore, the partial standing system cannot predict the end-of-season points total and is thus restricted purely to estimating final league position. Furthermore, because the partial standings do not take into account the relative strength of the opposition faced in the matches played, it can be the case, particularly in early season, that the standings do not accurately reflect the relative strengths of the teams in the league. For example, if a team has only faced weak opposition early in the season, then its standing in the league may be ‘over-inflated’ compared with that of other teams who have played matches against stronger opponents. Consequently, for teams with an ‘over-inflated’ league position, the partial standings may not be a good predictor of end-of-season league position. Indeed, this is one of the main reasons why the various sport ranking systems [3–6, 11–13, 35] were developed, so that a true indication of the relative strength of teams might be acquired for any point in time. In soccer, ranking systems have been widely used to compare the relative strengths of international teams [11, 36–38] and predict the outcome of matches in knock-out competitions [39] and leagues [40]. With respect to the ranking of international teams, Elo’s rating system appears to be particularly accurate [11]. Indeed, Elo’s method has been adopted by *Fédération Internationale de Football Association* (FIFA) to rank international teams in both men’s [41] and women’s [11] soccer.

Perhaps the most important finding of our study is that the partial standings of all four English leagues behaved in a similar manner, irrespective of the quality of the teams involved, with the normalized tau distance conforming to a power law and the Spearman r-value obeying a logarithmic function. Indeed, as illustrated in Fig 4, the normalized tau distance curves (and Spearman r-value curves–not shown) for all the 24-team leagues behaved in an almost identical fashion, indicating that the dynamic behaviour of the partial standings is primarily dictated by the format of these leagues and the points system used, rather than the quality of the teams involved. The normalized tau distance curve for the Premier League, although similar to that of the other leagues, had a slightly deeper profile indicating that the team positions tended to become ‘fixed in position’ earlier in the season than was the case with the other leagues. These findings are supported by those of Shin et al. [30], who in addition to obtaining the same results to us for Premier League seasons 2011–12 and 2012–13, also observed similar results for the NFL for seasons 2012 and 2013. As such, this implies that the partial standing dynamic may be ‘hard wired’ into the league itself and independent of the teams participating in the competition. Furthermore, the fact that Shin et al. observed similar findings for both the Premier League and the NFL suggests that this dynamic might be ‘hard wired’ into all sporting leagues, irrespective of the sport involved. While at this stage, any comparison between sports must remain a matter of conjecture, the fact that the shape of the normalized tau distance graph for the 20-team Premier League (Fig 4) so closely resembles those produced by the 24-team leagues, strongly suggests that all traditional soccer leagues share similar partial standing dynamics, irrespective of the actual number of teams involved and the quality of those teams. If this is the case, then it raises important questions regarding the ability of teams to affect their final league position when in mid-season. Our findings suggest that for all traditional soccer leagues, from a probabilistic point of view, the final league outcome becomes relatively fixed surprisingly early in the season, something that has been hitherto largely ignored.

In the English leagues each team normally plays all the other teams in the league once before Christmas and once after Christmas, with no account taken of the quality of the teams involved when scheduling fixtures. This format however, is not the same for all soccer leagues. For example in the Scottish Premier League, once every team has played each other three times the league splits into two halves for the final five games [42]. After this split, the top six teams play each other once, and the bottom six teams also play each other once. This raises intriguing questions as to whether or not changing the format of a soccer league might alter the dynamics of the partial standings. To address this issue we performed *post-hoc* analysis using data from the Premier League for season 2016–17, in which we restructured the league so that the top 10 pre-season ranked teams played the lower ranked teams in the first part of the season, while in the rest of the season the top 10 pre-season ranked teams played each other, and the lower ranked teams also played each other. As such, this represented an example of highly stratified competition as opposed to the traditional ‘round-robin’ format where teams of mixed ability play each other throughout the season. The results of this analysis (Fig 6) clearly show that restructuring the league had a substantial impact on the shape of the normalized tau distance curve. While both the real and restructured leagues still conformed to a power law, with the restructured league the tau distance decreased much more rapidly at the start of the season compared with the real league. This was because the strong teams had a lot of easy matches at the start of the season, which enabled them to firmly secure positions at the top of the league. Conversely, the weaker teams, faced with many difficult matches early in the season, found it hard to escape from the ‘foot of the table’. Consequently, the restructured league table became polarized much more quickly than was the case with the real league. In Fig 6 the error bars (±2 SDs) for the shuffled leagues give an indication of the spread of the tau distance curves that might occur if the fixtures were randomly reordered. As such, this indicates the likely limits of the changes in tau distance dynamics that are achievable simply by reordering the fixtures. This is well illustrated in Fig 6 where it can be seen that despite the fact that the restructured league is highly stratified and far from random, its tau distance curve lies entirely within the range of shuffled leagues. From this it can be concluded that, while it may be possible to alter the dynamics to a league to a certain extent by re-ordering the fixtures, the underlying tau distance trend will still conform to a power law.

While our findings, may be of interest to fans, pundits, and all those involved in the betting industry, perhaps they are of greater relevance to those involved in: (i) the management of soccer clubs; and (ii) the administration and governance of soccer leagues, many of whom may not realize that mathematical laws strongly influence the outcome of all soccer leagues. In particular, our findings suggest that timing is an issue of utmost importance, with major decisions regarding the acquisition of new players, or the ‘hiring and firing’ of managers, best undertaken earlier, rather than late, in the season—something highlighted by the results in Tables 7–10, where for example the chance of the bottom team in the Premier League being relegated, increases from 54.6% to 86.3% between rounds 10 to 30. By taking prudent remedial action early in the season, clubs appear to have a much better chance of avoiding relegation, rather than if they wait until much later before they act, as often occurs in English soccer when teams are facing the threat of relegation. Furthermore, even if the chance of being relegated cannot be substantially altered mid-way through the season, given the large financial implications associated with relegation [43], and the considerable costs associated with changing managers and other backroom staff part-way through a season [44], it is important for clubs to be able to make a realistic prediction of final league position as soon as possible, so that appropriate damage limitation measures can be taken well in advance. As such, knowing that the dynamic behaviour of soccer league standings conforms to a power law similar to those shown in Fig 3, may be of great benefit to all those involved in the planning and management of soccer clubs.

Our finding that partial standings in all soccer leagues appear to conform to the same dynamic should also be of interest to all those involved the administration and governance of soccer leagues, as well as the television companies who own league broadcasting rights. Although often forgotten, professional soccer is part of the entertainment industry, with player’s wages ultimately paid by the fans who watch matches either live or on television [45]. All income to soccer clubs, be it via the turn-styles, the television companies, or merchandising, ultimately relies on keeping the fans and viewers interested in the competition. Therefore, it becomes important to ensure that league competitions remain exciting at every stage until their final completion, so that interest can be maintained and revenue maximized. Unlike other branches of the entertainment industry, the outcome of sporting events is relatively uncertain, and it is this uncertainty that engenders excitement amongst fans [46] and drives the sports betting industry. Quirk and Fort [47] identified uncertainty over the outcome of individual matches, and uncertainty over the outcome of a particular season, as two distinct entities, both of which need to be accommodated if fan interest and excitement is to be maintained. While the outcome of individual soccer matches is known to exhibit a high degree of uncertainty [48, 49], our findings show that soccer leagues have an inherent tendency toward certainty as the season progresses, with the possibility that excitement may become compromised and the entertainment value of the ‘product’ depleted. Given the huge financial sums involved (e.g. in 2016 Sky Sports paid >£4 billion for the broadcasting rights to the Premier League [50]), broadcasters are particularly keen to ensure that their product remains ‘fresh and exciting’ to the very end the season. This can be a challenge towards the end of the season when matches involving mid-table teams can be relatively meaningless affairs. In an attempt to promote uncertainty and maintain end of season interest in the competition, some sporting leagues have incorporated a play-off system, although the structure of these varies considerably between sports. For example, in 1987 the English Football League introduced play-offs in order to decide, in part, promotion issues. By comparison, the English Rugby Super League and Rugby Union Premiership introduced play-offs with a grand final (in 1998 and 2002 respectively) to decide the outright winners of their respective competitions, irrespective of who actually finished top of the league. To purists, keen on traditional leagues, these adaptations may appear unfair. However, in the light of our findings, the strategy of artificially promoting end of season uncertainty appears to have considerable merit with regard to promoting interest in the competition and maximizing revenues. Having said this, any changes in the format of soccer leagues need to be carefully considered before implementation due to the risk of unintended side effects. For example, consider the case of the restructured Premier League format highlighted in the *post-hoc* analysis. While this two-stage format would produce more ‘high-stakes’ matches at the end of season, both at the top and the bottom of the league, the tau distance curves in Fig 6 tell us that the league would polarize much more quickly than would be the case with the traditional ‘round-robin’ format. Consequently, much of the uncertainty associated with the league would be quickly filtered out–something that might affect audience attendance figures. Furthermore, forcing the weaker teams, especially recently promoted teams, to play much stronger teams at the start of the season might have a profoundly demoralizing effect on both players and fans alike.

One interesting feature of our study relates to the fact that we took no consideration of the mid-season player transfer window that occurs from 1st– 31^st^ January in England, or of any changes in team management that occurred part way through the season, preferring instead to focus solely on the partial standing data. Soccer clubs frequently change manager mid-season (by the end of 2016–17, the average number of managerial changes each season in the Premier League stood at 8.84 [51]), generally in the hope that hiring a new manager will improve a club’s fortune. While the impact of managerial changes on league standing is beyond the scope of the present study, we note that whatever the changes that occurred in the 22 seasons studied, they did not alter the general dynamics of the partial standings or greatly inhibit their ability to predict final league position. As such, our findings appear to support those of others who have investigated the impact of managerial changes. For example, ter Weel [52] analyzed managerial turnover across 18 seasons (1986–2004) of the Dutch premier division, comparing teams who sacked their manager when things were not going well, with those who did not sack their manager when faced with a similar slump in form. He found that while changing a manager during a crisis improved results in the short-term, in the long-term it had little impact on season outcome, and that not changing the manager was just as likely to achieve the same result. Heuer et al. [53] who investigated the German Bundesliga, came to a similar conclusion, finding that managerial turnover has little effect of overall team performance beyond that of short-term improvement. If, as our results suggest, the outcome of soccer leagues is strongly influenced by mathematical laws, then it is perhaps not surprising that these researchers found managerial changes to have minimal impact on long-term outcomes.

While in the present study we evaluated the ability of partial standings to predict the end-of-season rankings, we did not consider the impact of past performance in previous seasons on this metric. Given that final league position in the previous season can often be a good indicator of team performance in the next season [54], it is recommended that future work in this field, investigate the extent to which past performance in previous seasons influences the league standings in future seasons. In addition, more work needs to be undertaken on the effect of different competition structures on league dynamics. While we have been able to show, in a limited way, that it is possible to alter the tau distance dynamics in soccer leagues by stratifying the competition, much more work will be needed to establish whether or not this effect has general applicability, or indeed to identify the optimum league format for any given competition.

## Conclusions

The principal finding of our study is that dynamic behaviour of the partial standings in all four English leagues is very similar, irrespective of the quality of the teams involved, with the change in normalized tau distance strongly conforming to a power law. As such, this suggests that the dynamics of all traditional soccer leagues are similar and primarily dictated by the format of the league and the points system used, rather than anything to do with the specific teams. While it appears to be possible to change the dynamics of the league to a limited extent though restructuring the competition, this does not appear to alter the underlying trend in the tau distance metric, which still conforms to a power law no matter the order in which fixtures are played. These findings suggest that individual teams have only limited ‘free will’ regarding the end-of-season outcome because the mathematics of the competition increasingly constrains ‘cross-over’ events in league table position as the season progresses. Indeed, by approximately round 10 of competition much of the variance in the end-of-season league position appears to be explained. Partial standings can therefore be considered as a reasonable predictor of final league position, and as such appear to have considerable potential as a planning and management tool in professional soccer. In particular, our findings suggest that if a change in team manager is required, then it is better that it be done early, rather than waiting until much later in the season, when the ability to influence the end-of-season outcome is much more limited.

## References

[1] Davis C. Premier League 2017 prize money: How much your club is in line to earn this season?. The Telegraph [Internet]. 2017 4th January 2018. http://www.telegraph.co.uk/football/2017/05/16/premier-league-2017-prize-money-much-club-line-earn-season/.

[2] McCourt I. Parma relegated to Serie D after failing to find a new owner. The Guardian [Internet]. 2015. https://www.theguardian.com/football/2015/jun/22/parma-relegated-serie-d-fail-new-owner.

[3] Colley WN. Colley’s bias free college football ranking method: The Colley matrix explained. Princeton University, Princeton 2002.

[4] Langville AN, Meyer CD. Who’s # 1?: the science of rating and ranking. Princeton: Princeton University Press; 2012.

[5] Beggs CB, Shepherd SJ, Emmonds S, Jones B. A novel application of PageRank and user preference algorithms for assessing the relative performance of track athletes in competition. PLoS One. 2017;12(6):e0178458 10.1371/journal.pone.0178458 . 28575009 PMC5456068

[6] Keener JP. The Perron-Frobenius theorem and the ranking of football teams. SIAM review. 1993;35(1):80–93.

[7] Dayaratna KD, Miller SJ. The pythagorean won-loss formula and hockey. The Hockey Research Journal. 2013;2012/13:193–209.

[8] Caro CA, Machtmes R. Testing the utility of the pythagorean expectation formula on division one college football: an examination and comparision to the Morey model. Journal of Business & Economic Research. 2013;11(12):537–42.

[9] Bradley RA, Terry ME. Rank analysis of incomplete block designs: I. the method of paired comparisons Biometrika. 1952;39(3/4):324–45.

[10] Mchale I, Morton A. A Bradley-Terry type model for forecasting tennis match results. International Journal of Forecasting. 2011;27(2):619–30.

[11] Lasek J, Szlavik Z, Bhulai S. The predictive power of ranking systems in association football. International Journal of Applied Pattern Recognition. 2013;1(1):27–46.

[12] Agresti A. An introduction to categorical data analysis: Wiley; 2018.

[13] Van Haaren J, Davis J, editors. Predicting the final league tables of domestic football leagues. Proceedings of the 5th international conference on mathematics in sport; 2015.

[14] Burer S. Robust rankings for college football. Journal of Quantitative Analysis in Sports. 2012;8(2):1–22.

[15] Chartier TP, Harris J, Hutson KR, Langville AN, Martin D, Wessell CD. Reducing the Effects of Unequal Number of Games on Rankings. IMAGE-The Bulletin of the International Linear Algebra Society. 2014;52(1).

[16] Govan AY, Meyer CD, editors. Ranking national football league teams using google’s pagerank. AA Markov Anniversary Meeting; 2006; Charleston: Boson Books.

[17] Balreira EC, Miceli BK, Tegtmeyer T. An Oracle method to predict NFL games. Journal of Quantitative Analysis in Sports. 10(2):183–96.

[18] Mease D. A penalized maximum likelihood approach for the ranking of college football teams independent of victory margins The American Statistician. 2003;57(4):241–8.

[19] Tsokos A, Narayanan S, Kosmidis I, Baio G, Cucuringu M, Whitaker G, et al Modeling outcomes of soccer matches Machine Learning. 2018:1–19.

[20] van der Zaan T. Predicting the outcome of soccer matches in order to make money with betting. Rotterdam: Erasmus University Rotterdam; 2017.

[21] Heuer A, Rubner O. How does the past of a soccer match influence its future? Concepts and statistical analysis. PloS one. 2012;7(11):e47678 10.1371/journal.pone.0047678 23226200 PMC3511508

[22] Heuer A, Muller C, Rubner O. Soccer: Is scoring goals a predictable Poissonian process? Europhysics Letters. 2010;89:38007.

[23] Saraiva EF, Suzuki AK, Filho CAO, Louzada F. Predicting football scores via Poisson regression model: applications to the National Football League. Communications for Statistical Applications and Methods. 2016;23(4):297–319.

[24] Constantinou AC. Dolores: A model that predicts football match outcomes from all over the world Machine Learning. 2018:1–27.

[25] Razali N, Mustapha A, Yatim FA, Ab Aziz R, editors. Predicting Football Matches Results using Bayesian Networks for English Premier League (EPL). IOP Conference Series: Materials Science and Engineering; 2017: IOP Publishing.

[26] Louzada F, Suzuki AK, Salasar LEB. Predicting match outcomes in the English Premier League: Which will be the final rank? Journal of Data Science. 2014;12:235–54.

[27] Jurman G. Seasonal linear predictivity in national football championships. arXiv:151106262v1 [statAP] 19 Nov 2015. 2015. 10.1089/big.2018.0076 30888213

[28] Baker LB, Stofan JR, Hamilton AA, Horswill CA. Comparison of regional patch collection vs. whole body washdown for measuring sweat sodium and potassium loss during exercise. J Appl Physiol (1985). 2009;107(3):887–95. 10.1152/japplphysiol.00197.2009 . 19541738

[29] Lasek J, Gagolewski M. The efficacy of league formats in ranking teams. Statistical Modelling. 2018;18(5–6):411–35.

[30] Shin S, Ahnert SE, Park J. Ranking competitors using degree-neutralized random walks. PLoS One. 2014;9(12):e113685 10.1371/journal.pone.0113685 . 25517977 PMC4269436

[31] opisthokonta.net. R functions for soccer league tables and result matrix. opisthokontanet [Internet]. 2012 4th January 2018. http://opisthokonta.net/?p=18.

[32] Fagin R, Kumar R, Sivakumar D. Comparing top k lists. SIAM Journal on discrete mathematics. 2003;17(1):134–60.

[33] Borchers HW. Pracma: practical numerical math functions. R package version. 2015;1(3).

[34] Bunker RP, Thabtah F. A machine learning framework for sport result prediction Applied Computing and Informatics. 2017.

[35] Massey K. Statistical models applied to the rating of sports teams. Bluefield College 1997.

[36] World football Elo ratings [Internet]. 1997 [cited 10th March 2017]. http://www.eloratings.net/.

[37] eloratings.net. World Football Elo Ratings [12th July 2019]. http://www.eloratings.net/about.

[38] Lazova V, Basnarkov L. PageRank Approach to Ranking National Football Teams. arXiv preprint arXiv:150301331. 2015.

[39] Suzuki K, Ohmori K. Effectiveness of FIFA/Coca-Cola World Ranking in predicting the results of FIFA World Cup finals. Football Science. 2008;5:18–25.

[40] masseyratings.com. Massey Ratings [updated 12th July 2019]. https://www.masseyratings.com/index.htm.

[41] Price S. How FIFA’s New Ranking System Will Change International Soccer. Forbes [Internet]. 2018 12th July 2019. https://www.forbes.com/sites/steveprice/2018/06/11/how-fifas-new-ranking-system-will-change-international-soccer/#18864e86c412.

[42] BBC. Guide to the SPL split 2001 [9th June 2019]. http://news.bbc.co.uk/sport1/hi/football/scot_prem/1251646.stm.

[43] Switzer A. The cost of relegation from the Premier League. The Telegraph. 2011;23.

[44] Flint SW, Plumley DJ, Wilson RJ. You don’t know what you’re doing! The impact of managerial change on club performance in the English Premier League. Managing Leisure. 2014;19(6):390–9.

[45] Blumrodt J, Desbordes M, Bodin D. Professional football clubs and corporate social responsibility. Sport, Business and Management: An International Journal. 2013;3(3):205–25.

[46] Elias N, Dunning E. Quest for excitement: sport and leisure in the civilising process. Oxford: Blackwell; 1986.

[47] Quirk J, Fort RD. Pay dirt: the business of professional team sports. Princeton, N.J.: Princeton University Press; 1992.

[48] Forrest D, Simmons R. Outcome uncertainty and attendance demand in sport: the case of English soccer. Journal of the Royal Statistical Society: Series D (The Statistician). 2002;51(2):229–41.

[49] Buraimo B, Simmons R. Do sports fans really value uncertainty of outcome? Evidence from the English Premier League. International Journal of Sport Finance. 2008;3(3).

[50] Rumsby B. Premier League TV deal: Sky Sports break bank to dominate £5.136bn contract. The Telegraph [Internet]. 2015 4th January 2018. http://www.telegraph.co.uk/sport/football/11403761/Premier-League-TV-deal-Sky-Sports-break-bank-to-dominate-5.136bn-contract.html.

[51] Johnson N. Premier League is 25 years old: Facts and figures behind the first quarter-century. BBC news [Internet]. 2017 4th January 2018. http://www.bbc.co.uk/sport/football/40704646.

[52] ter Weel B. Does Manager Turnover Improve Firm Performance? Evidence from Dutch Soccer, 1986–2004. De Economist. 2011;159(3):279–303.

[53] Heuer A, Muller C, Rubner O, Hagemann N, Strauss B. Usefulness of dismissing and changing the coach in professional soccer. PLoS One. 2011;6(3):e17664 10.1371/journal.pone.0017664 . 21445335 PMC3062539

[54] Sumpter D. Soccermatics: mathematical adventures in the beautiful game: Bloomsbury Publishing; 2016.

